# Direct maternal morbidity and the risk of pregnancy-related deaths, stillbirths, and neonatal deaths in South Asia and sub-Saharan Africa: A population-based prospective cohort study in 8 countries

**DOI:** 10.1371/journal.pmed.1003644

**Published:** 2021-06-28

**Authors:** Fahad Aftab, Imran Ahmed, Salahuddin Ahmed, Said Mohammed Ali, Seeba Amenga-Etego, Shabina Ariff, Rajiv Bahl, Abdullah H. Baqui, Nazma Begum, Zulfiqar A. Bhutta, Godfrey Biemba, Simon Cousens, Vinita Das, Saikat Deb, Usha Dhingra, Arup Dutta, Karen Edmond, Fabian Esamai, Amit Kumar Ghosh, Peter Gisore, Caroline Grogan, Davidson H. Hamer, Julie Herlihy, Lisa Hurt, Muhammad Ilyas, Fyezah Jehan, Mohammed Hamad Juma, Michel Kalonji, Rasheda Khanam, Betty R. Kirkwood, Aarti Kumar, Alok Kumar, Vishwajeet Kumar, Alexander Manu, Irene Marete, Usma Mehmood, Nicole Minckas, Shambhavi Mishra, Dipak K. Mitra, Mamun Ibne Moin, Karim Muhammad, Sam Newton, Serge Ngaima, Andre Nguwo, Muhammad Imran Nisar, John Otomba, Mohammad Abdul Quaiyum, Sophie Sarrassat, Sunil Sazawal, Katherine E. Semrau, Caitlin Shannon, Vinay Pratap Singh, Sajid Soofi, Seyi Soremekun, Atifa Mohammed Suleiman, Venantius Sunday, Thandassery R. Dilip, Antoinette Tshefu, Yaqub Wasan, Kojo Yeboah-Antwi, Sachiyo Yoshida, Anita K. Zaidi, on behalf of The Alliance for Maternal and Newborn Health Improvement (AMANHI) maternal morbidity study group

**Affiliations:** 1 Centre for Public Health Kinetics (CPHK), New Delhi, Delhi, India; 2 Centre of Excellence in Women and Child Health, The Aga Khan University, Karachi, Pakistan; 3 Projahnmo Research Foundation, Dhaka, Bangladesh; 4 Public Health Laboratory-IdC, Pemba Island, Zanzibar, United Republic of Tanzania; 5 Kintampo Health Research Centre, Ghana Health Service, Kintampo, Ghana; 6 Department of Maternal Newborn Child and Adolescent Health and Ageing, World Health Organization, Geneva, Switzerland; 7 Department of International Health, Johns Hopkins Bloomberg School of Public Health, Baltimore, Maryland, United States of America; 8 National Health Research Authority, Ministry of Health, Lusaka, Zambia; 9 London School of Hygiene & Tropical Medicine, London, United Kingdom; 10 Department of Gynaecology & Obstetrics, King George’s Medical University, Lucknow, India; 11 King’s College London, London, United Kingdom; 12 Department of Child Health and Pediatrics, Eldoret, Moi University, Kenya; 13 Government of India, New Delhi, India; 14 Ariadne Labs, Harvard T.H Chan School of Public Health, Brigham and Women’s Hospital, Boston, Massachusetts, United States of America; 15 Department of Global Health, Boston University School of Public Health, Boston, Massachusetts, United States of America; 16 Section of Infectious Diseases, Department of Medicine, Boston Medical Center, Boston, Massachusetts, United States of America; 17 Department of Pediatrics, Boston University School of Medicine, Boston, Massachusetts, United States of America; 18 Cardiff University School of Medicine, Cardiff, United Kingdom; 19 Department of Paediatrics and Child Health, The Aga Khan University, Karachi, Pakistan; 20 Department of Community Health, Kinshasa School of Public Health, Kinshasa, Demographic Republic of Congo; 21 Community Empowerment Lab, Shivgarh, India; 22 Government of Uttar Pradesh, India; 23 School of Public Health, University of Ghana, Accra, Ghana; 24 Liverpool School of Tropical Medicine, Liverpool, United Kingdom; 25 Department of Statistics, Lucknow University, Lucknow, India; 26 North South University, Dhaka, Bangladesh; 27 Kwame Nkrumah University of Science & Technology, Kumasi Ghana; 28 Harvard Medical School, Department of Medicine, Boston, Massachusetts, United States of America; 29 Brigham and Women’s Hospital, Division of Global Health Equity, Boston, Massachusetts, United States of America; 30 CARE USA, Atlanta, United States of America

## Abstract

**Background:**

Maternal morbidity occurs several times more frequently than mortality, yet data on morbidity burden and its effect on maternal, foetal, and newborn outcomes are limited in low- and middle-income countries. We aimed to generate prospective, reliable population-based data on the burden of major direct maternal morbidities in the antenatal, intrapartum, and postnatal periods and its association with maternal, foetal, and neonatal death in South Asia and sub-Saharan Africa.

**Methods and findings:**

This is a prospective cohort study, conducted in 9 research sites in 8 countries of South Asia and sub-Saharan Africa. We conducted population-based surveillance of women of reproductive age (15 to 49 years) to identify pregnancies. Pregnant women who gave consent were include in the study and followed up to birth and 42 days postpartum from 2012 to 2015. We used standard operating procedures, data collection tools, and training to harmonise study implementation across sites. Three home visits during pregnancy and 2 home visits after birth were conducted to collect maternal morbidity information and maternal, foetal, and newborn outcomes. We measured blood pressure and proteinuria to define hypertensive disorders of pregnancy and woman’s self-report to identify obstetric haemorrhage, pregnancy-related infection, and prolonged or obstructed labour. Enrolled women whose pregnancy lasted at least 28 weeks or those who died during pregnancy were included in the analysis. We used meta-analysis to combine site-specific estimates of burden, and regression analysis combining all data from all sites to examine associations between the maternal morbidities and adverse outcomes.

Among approximately 735,000 women of reproductive age in the study population, and 133,238 pregnancies during the study period, only 1.6% refused consent. Of these, 114,927 pregnancies had morbidity data collected at least once in both antenatal and in postnatal period, and 114,050 of them were included in the analysis. Overall, 32.7% of included pregnancies had at least one major direct maternal morbidity; South Asia had almost double the burden compared to sub-Saharan Africa (43.9%, 95% CI 27.8% to 60.0% in South Asia; 23.7%, 95% CI 19.8% to 27.6% in sub-Saharan Africa). Antepartum haemorrhage was reported in 2.2% (95% CI 1.5% to 2.9%) pregnancies and severe postpartum in 1.7% (95% CI 1.2% to 2.2%) pregnancies. Preeclampsia or eclampsia was reported in 1.4% (95% CI 0.9% to 2.0%) pregnancies, and gestational hypertension alone was reported in 7.4% (95% CI 4.6% to 10.1%) pregnancies. Prolonged or obstructed labour was reported in about 11.1% (95% CI 5.4% to 16.8%) pregnancies. Clinical features of late third trimester antepartum infection were present in 9.1% (95% CI 5.6% to 12.6%) pregnancies and those of postpartum infection in 8.6% (95% CI 4.4% to 12.8%) pregnancies. There were 187 pregnancy-related deaths per 100,000 births, 27 stillbirths per 1,000 births, and 28 neonatal deaths per 1,000 live births with variation by country and region. Direct maternal morbidities were associated with each of these outcomes.

**Conclusions:**

Our findings imply that health programmes in sub-Saharan Africa and South Asia must intensify their efforts to identify and treat maternal morbidities, which affected about one-third of all pregnancies and to prevent associated maternal and neonatal deaths and stillbirths.

**Trial registration:**

The study is not a clinical trial.

## Author summary

**Why was this study done?:**

- Estimates of severe direct maternal morbidity are largely based on hospital-based studies with inconsistent definitions and varying selection criteria.
- Limited data are available from South Asia and sub-Saharan Africa, the 2 regions with the highest maternal and newborn morbidity and mortality, where a larger proportion of births occur at home.

**What did the researchers do and find?:**

- We collected data on maternal morbidities from a cohort of women in the community in multiple sites in sub-Saharan Africa and in South Asia.
- Out of a cohort of >114,000 women, we found that about 1 in 3 women suffer a maternal morbidity, which was notably higher than the previously reported data.
- We found that the prevalence of preeclampsia and eclampsia was about 1%, which was lower than previously reported.
- About 11% of women reported having prolonged or obstructed labour, which was somewhat higher than published estimates.
- The burden of pregnancy-related infection in postpartum period was higher in South Asia than in sub-Saharan Africa.
- This is the first study, to our knowledge, to describe the burden of antepartum haemorrhage and late antepartum infection and to clearly demonstrate the association of direct maternal morbidity with adverse pregnancy outcomes.

**What do these findings mean?:**

- Higher burden of direct maternal morbidity and its association with adverse outcomes highlights the need for improving health of women and mothers, including promotion of preconception health and nutrition, and high-quality antepartum, intrapartum and postpartum care.

## Introduction

Safe motherhood programmes have largely focused on reduction of maternal mortality [1]. Inadequate attention has been paid to the morbidities that women experience during the pregnancy, intrapartum, and postpartum period. For every woman who dies of a maternal cause, an estimated 20 to 30 women experience acute or chronic morbidity with substantial impact on physical, psychological, social, and economic outcomes [2–5]. According to current WHO estimates, approximately 15% of all pregnant women or about 20 million women annually experience acute severe obstetric complications, including haemorrhage, obstructed or prolonged labour, preeclampsia or eclampsia, puerperal sepsis, and septic abortion [6]. These conditions affect the health of the foetus and newborn, in addition to the woman. For example, the risk of perinatal mortality increases with placental abruption, ruptured uterus, systemic infections/sepsis, preeclampsia, eclampsia, and severe anaemia [7]. Moreover, women whose first pregnancy ends in stillbirth or is followed by death of the neonate is at increased risk of experiencing the same outcome in her subsequent pregnancy [8].

Efforts to develop effective programmes and offer appropriate services to address maternal morbidity in low- and middle-income countries (LMICs) have been undermined by the paucity of reliable data on maternal morbidity and its sequelae at the population level [9]. First, maternal morbidity estimates have been constrained by the use of inconsistent definitions and measurement methods of morbidities and their severity. A systematic literature review and meta-analysis of severe maternal morbidity found its prevalence to vary between 0.05% and 15% of hospitalised women depending on the definition used [10]. Second, studies that aimed to quantify the burden of maternal morbidity based their prevalence estimates on facility-based data, which may not reflect the true burden at the population level particularly in population where a sizable proportion of deliveries occurring at home. The largest such study examined maternal morbidity in women attending health facilities in 29 countries from Africa, Asia, Latin America, and the Middle East [11]. It was found that 7.3% of women had potentially life-threating conditions and that 1% developed a severe maternal outcome (defined as maternal death or near miss). However, unbiased and accurate diagnosis of maternal morbidity in population-level studies becomes difficult under survey conditions without clinical examination, laboratory reports, or medical records [12]. Point-in-time estimates from cross-sectional surveys of maternal morbidity can be unreliable [13,14]. Not surprisingly, surveys found that 70% of women or more report signs or symptoms of pregnancy-related complications [12,15,16]. Therefore, clear need exists to generate reliable population-based estimates of maternal morbidity in LMICs using robust epidemiological methods.

The Alliance for Maternal and Newborn Health Improvement (AMANHI) study was designed to provide these data from large community-based cohorts of pregnant and postpartum women in 9 sites in 8 countries across sub-Saharan Africa and South Asia. For 4 direct maternal morbidities (obstetric haemorrhage, hypertensive disorders of pregnancy, pregnancy-related infection, and prolonged or obstructed labour), we aimed to (1) ascertain their prevalence across sites and region and (2) examine their associations with pregnancy-related death of women, stillbirth, and neonatal death.

## Methods

### Study overview

The AMANHI maternal morbidity study design and objectives have been described previously (S1 Text) [17]. In brief, this population-based, cohort study of pregnant women was conducted in Bangladesh (Sylhet), India (Uttar Pradesh), and Pakistan (Karachi and Matiari, both Sindh) in South Asia; and Democratic Republic of Congo (Equateur), Ghana (Brong Ahafo), Kenya (Western Province), Tanzania (Pemba), and Zambia (Southern Province) in sub-Saharan Africa. Data were collected from 2012 to 2015 and built on the platform of ongoing community-based research. The total population across sites was nearly 4 million with more than 735,000 women of reproductive age in the surveillance areas. These sites were predominantly in rural settings and represented a range of maternal and newborn mortality.

Through a harmonisation process, a core protocol, core variable table, and schedule of visits were agreed upon by WHO and site principal investigators. In all sites except Zambia, trained fieldworkers made home visits to all women of reproductive age residing in the study area every 2 to 3 months to obtain consent and interview them and ask them if they were pregnant, and if they were pregnant, take consent for participation in the study, and collect baseline data. During home visits, fieldworkers use a variety of methods to identify pregnant women. These include direct disclosure by women or eliciting information on missed menstrual periods from women’s LMPs. When unsure, women in Bangladesh, Pakistan (Karachi and Matiari, Sindh), India (UP), and Tanzania (Pemba) had the option to request a urine pregnancy test to confirm pregnancies. In Zambia, recruitment was facility based; this strategy was also population based as over 96% of women in the study area attend antenatal care clinics during pregnancy [18]. Pregnant women gave consent prior to enrolment in the cohort and to have their pregnancy followed through the end of the postpartum period (after 42 days’ postpartum).

We conducted home visits at 5 time points: at 6 months’ pregnancy (24 to 28 weeks, or at the time of identification of pregnancy, if later), at around 8 months’ pregnancy (32 to 37 weeks), at 9 months’ pregnancy (38 to 40 weeks’ pregnancy), in the first postpartum week, and at the end of the postpartum period (at 7 to 11 postpartum weeks). These included blood pressure (Microlife WatchBP Home A BP3MX1-3, Widnau, Switzerland) and proteinuria (Uristix by Siemens, Gujarat, India) measurements were performed at each visit. These study materials were procured from a common source.

At enrolment, we collected data on baseline sociodemographic information and previous medical and obstetric history. At home visits during pregnancy, data were collected on general health, pregnancy-related morbidities, and care seeking; in addition, data were extracted from the antenatal card, including the number and timing of visits, gestational age assessment, and care and treatment received. At the first visit, morbidities were assessed from the beginning of pregnancy; at subsequent visits, morbidities assessed since the previous visit. For each morbidity, we assessed the time of onset, severity, and any interventions received and where from. At the first postpartum visit after birth, in addition to data on morbidities and care seeking, data on labour, delivery, and immediate postpartum complications were captured for both the mother and the neonate, as well as data on infant sex, birth weight, and feeding patterns were collected.

Local and institutional ethics committees from all 9 sites approved the AMANHI study protocols. The Ethics Review Committee of WHO also approved the protocol (RPC 532).

### Data management and quality assurance

Study staff underwent rigorous training for data collection. An independent team of study supervisors conducted random spot checks of all workers once a month and monitored quality of activities. Six monthly training sessions were held to standardise measurement of blood pressure and proteinuria.

The 9 sites used standardised questionnaires with a defined set of core variables, and these questionnaires were completed by interview, examination, anthropometry, or laboratory analyses. Data managers within the sites conduct inter-database checks to reconcile and synchronise data from various forms using the woman’s unique study ID as the link. Every 3 months, sites transferred backup data to a dedicated server at WHO/MCA for external quality control and storage. WHO undertook site monitoring, including observation of data collection, in all sites twice every year.

### Outcomes and definitions

Women were included in the cohort analysis if they had at least one home visit during pregnancy and one home visit after birth in the postnatal period or if they died in pregnancy. We excluded pregnancies not reaching 28 weeks in the analyses to assess burden of maternal morbidities and association of maternal morbidities with adverse outcomes. We assessed the following direct maternal morbidities: obstetric haemorrhage (antepartum and postpartum), hypertensive disorders of pregnancy (preeclampsia and eclampsia, hypertension only), pregnancy-related infection (late antepartum infection, postpartum infection), and prolonged or obstructed labour. We measured incidence and timing of stillbirths, neonatal deaths, and pregnancy-related deaths.

Antepartum haemorrhage was defined using women’s self-report of bleeding from vagina occurring any time during pregnancy that wet her clothes (to exclude minor spotting).

Severe postpartum haemorrhage is usually defined based on greater than 1,000 ml blood loss after birth of a baby. Measurement of blood loss after birth was not possible in our study. In the community-based study especially for deliveries that take place at home, it was not possible to quantify the amount of blood lost. We therefore used pragmatic definitions and defined severe postpartum haemorrhage as self-report of bleeding in the first week after birth that resulted in loss of consciousness or required treatment by blood transfusion, hysterectomy, or other surgery.

We categorised women as having hypertensive disorders of pregnancy based on objective measures of blood pressure and dipstick-measured proteinuria during home visits in pregnancy or after birth. In line with ACOG guidelines [19], we defined preeclampsia as diastolic blood pressure ≥90 mm Hg and/or systolic blood pressure ≥140 mm Hg and proteinuria at the same visit, and eclampsia as preeclampsia accompanied by convulsions at the same visit or a subsequent visit.

We categorised women as having late antepartum or postpartum infection based on women’s self-report of having had fever or smelly discharge or pus pass from vagina. Late antepartum refers to the time from third trimester of pregnancy until delivery.

Women whose labour started ≥24 hours before delivery or who had cesarean delivery after initiation of labour on account of a “big baby,” “small pelvis,” abnormal lie, or ruptured/imminent rupture of the uterus were classified as having had prolonged or obstructed labour.

If woman died and information on morbidity was not available by home visit, we obtained information on maternal morbidity that woman had prior to death by verbal autopsy. The verbal autopsy was based on ICD-10 classification system [20].

Pregnancy-related death is a reflective of maternal deaths; however, the definition of pregnancy-related death includes all causes of deaths (obstetric and nonobstetric) including accidental or incidental causes during pregnancy till postpartum 42 days while maternal deaths exclude deaths from such causes.

Stillbirth was defined as a foetal death after 28 weeks gestation. Antepartum stillbirth was defined as foetal death occurring after 28 weeks of gestation and before the onset of labour. Intrapartum stillbirth was foetal deaths occurring after the onset of labour and before the delivery. Neonatal death was defined as the death of an infant during the first 28 days of life. Pregnancy-related death was defined as the death of a woman while pregnant or within 42 days of termination of pregnancy, irrespective of the duration and site of the pregnancy, from any cause.

### Statistical analysis

In our planning for the cohort, we expected to have 160,000 pregnancies in the study. This sample size would have been sufficient to estimate a prevalence of maternal morbidity of 2% with a relative precision of ±5% for each region (sub-Saharan Africa and South Asia). We ended up with 114,927 pregnancies in the cohort. This shortfall in the sample means that we can estimate a prevalence of maternal morbidity of 2% with relative precision ±6% for each region (sub-Saharan Africa and South Asia).

We summarised baseline data on household, maternal, and infant characteristics using means or medians for continuous data and proportions for categorical data. We summarised morbidity prevalence and incidence using proportions.

We estimated the burden of each maternal morbidity (as a proportion of deliveries affected), pregnancy-related deaths, stillbirth, and neonatal mortality for each site separately. To obtain regional and global summary estimates of burden, we combined site-specific estimates using random effects meta-analysis. We then used logistic regression to investigate the association between each maternal morbidity and the following outcomes: pregnancy-related deaths, stillbirths, and neonatal deaths. Each maternal morbidity outcome was analysed in a separate model. In each model, we adjusted for wealth quintile, educational attainment, maternal age, parity, multiple gestation, and site.

We used meta-analysis to combine site-specific estimates of burden (descriptive analyses), while we used regression analysis combining all of the data from all sites to examine associations between the maternal morbidities (now the explanatory variable, not the outcome) and pregnancy-related deaths, stillbirths, and neonatal deaths (the outcomes). In the latter analyses, site was included as a covariate. All *p*-values were two sided. Analyses was done using Stata 14.0 statistical software package [21]. Missing data were reported in the footnotes of each result table. We did not use any imputation methods for missing data.

## Results

### Study participants

**Fig 1:**
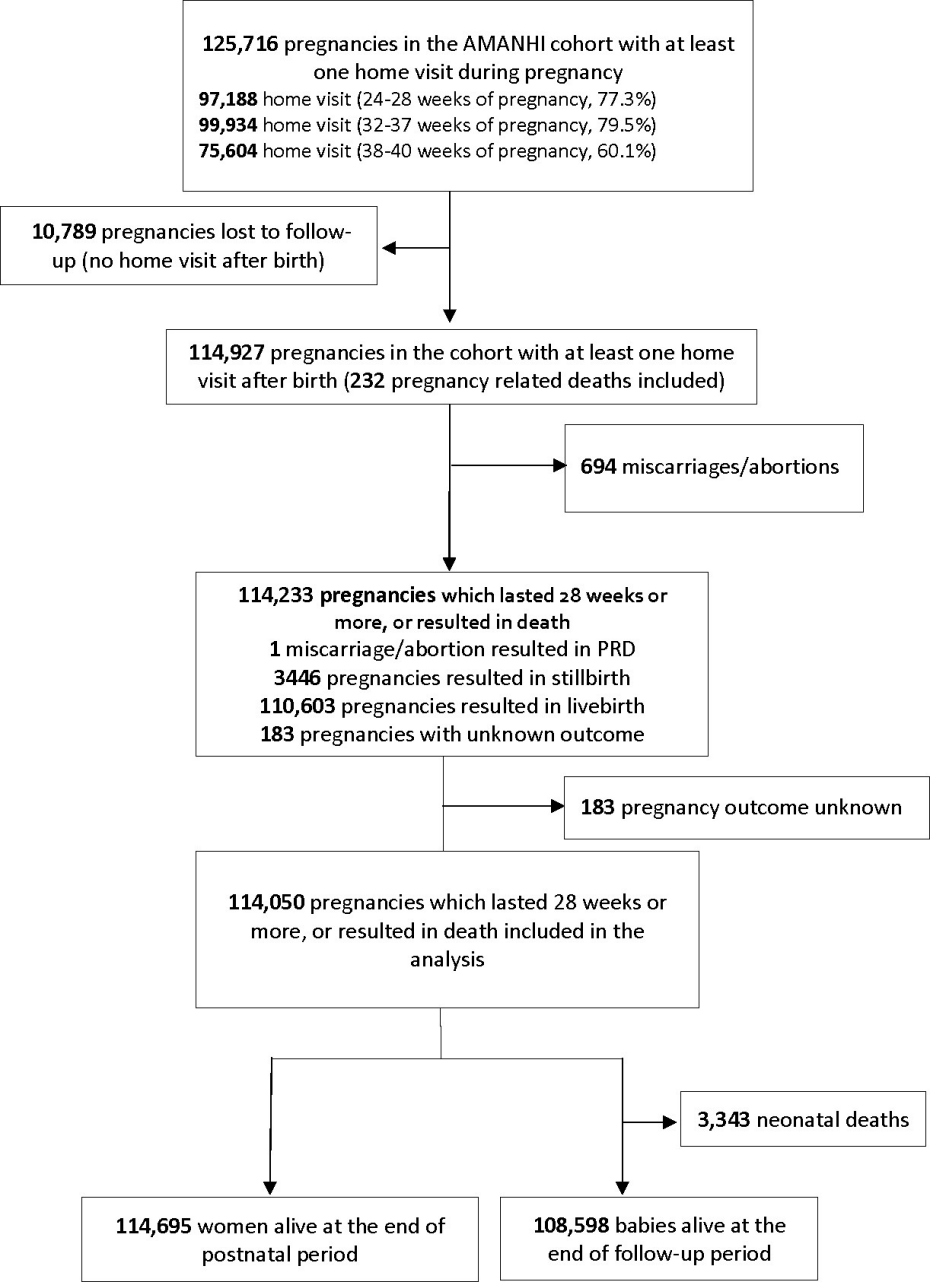
Flowchart of pregnant women enrolled in the AMANHI maternal morbidity study for all sites combined. AMANHI, Alliance for Maternal and Newborn Health Improvement; PRD, pregnancy-related death.

Across the 9 sites, 114,927 pregnancies were identified, and 114,050 of them were included in the analysis (Fig 1 and Table 1).

**Table 1:** Baseline characteristics of pregnant women enrolled in AMANHI maternal morbidity study.

Characteristics	Total	Bangladesh	DRC	Ghana	India Shivgarh	Kenya	Pakistan Karachi	Pakistan Matiari	Tanzania Pemba	Zambia
**Pregnancies in the cohort (*N*)**	114,927	11,367	7,031	12,834	35,049	8,528	3,686	12,59	16,854	6,985
Woman’s age, Median (IQR)	25 (22 to 30)	24 (20 to 27)	25 (20 to 29)	27 (22 to 32)	25 (22 to 27)	24 (20 to 28)	26 (22 to 30)	29 (25 to 33)	27 (22 to 32)	24 (20 to 30)
Woman’s age in category, *n* (%)										
15–19	10,312 (9.0)	1,945 (17.1)	1,174 (16.7)	1,490 (11.6)	397 (1.1)	1,616 (19.0)	299 (8.1)	220 (1.8)	1,469 (8.7)	1,702 (24.4)
20–29	73,368 (63.8)	7,408 (65.2)	3,673 (52.3)	6,839 (53.4)	29,580 (84.4)	5,039 (59.1)	2,185 (59.3)	6,309 (50.1)	8,985 (53.3)	3,350 (48.0)
30–39	27,610 (24.0)	1,906 (16.8)	1,445 (20.6)	3,970 (31.0)	4,852 (13.8)	1,561 (18.3)	1,126 (30.6)	5,626 (44.7)	5,471 (32.5)	1,653 (23.7)
>40	2,637 (2.3)	108 (1.0)	128 (1.8)	515 (4.0)	114 (0.3)	88 (1.0)	73 (2.0)	435 (3.5)	928 (5.5)	249 (3.6)
Missing	1,000 (0.9)	0	611 (8.7)	21 (0.2)	106 (0.3)	224 (2.6)	3 (0.1)	3 (0.0)	1 (0.0)	31 (0.4)
Woman attended school, *n* (%)	73,882 (66.8)	9,813 (89.5)	3,985 (71.2)	9,168 (71.6)	21,349 (61.7)	8,181 (99.4)	971 (26.5)	2,329 (18.5)	11,973 (76.7)	6,113 (93.8)
Women with previous livebirth*, *n* (%)	80,161 (71.2)	7,724 (67.8)	5,478 (82.2)	9,399 (73.4)	20,864 (59.7)	6,120 (73.9)	2,762 (75.3)	9,830 (78.1)	13,099 (83.9)	4,885 (73.8)
Number of previous livebirths^†^, Median (IQR)	2 (1 to 4)	2 (1 to 3)	3 (2 to 5)	2 (1 to 4)	2 (1 to 3)	2 (1 to 4)	3 (1 to 4)	3 (2 to 5)	4 (2 to 6)	3 (2 to 5)
Women with previous stillbirth*, *n* (%)	7,292 (8.7)	916 (11.2)	465 (8.4)	919 (9.1)	1,822 (8.3)	213 (3.4)	196 (6.8)	1,429 (13.8)	1,061 (8)	271 (5.5)
Women with previous miscarriage^†^, *n* (%)	16,249 (19.5)	1,704 (20.8)	786 (14.1)	2,295 (22.8)	4,303 (19.5)	403 (6.5)	792 (27.4)	2,726 (26.4)	3,003 (22.6)	237 (4.8)
Women with previous C section*, *n* (%)	3,076 (3.7)	231 (2.8)	59 (1.1)	525 (5.2)	816 (3.7)	89 (1.4)	132 (4.6)	875 (8.5)	294 (2.2)	55 (1.2)
Women with previous preterm birth*, *n* (%)	2,772 (3.3)	288 (3.5)	310 (5.6)	213 (2.1)	719 (3.3)	274 (4.4)	84 (2.9)	301 (2.9)	413 (3.1)	170 (3.5)
Household access to improved drinking water source^‡^, *n* (%)	82,754 (73.5)	5,481 (48.1)	229 (3.4)	10,539 (82.3)	33,640 (96.3)	2,492 (30.1)	2,221 (60.6)	12,402 (98.6)	13,934 (89.3)	1,816 (27.7)
Household access to toilet facility^#^, *n* (%)	16,960 (15.1)	4,153 (36.5)	87 (1.3)	320 (2.5)	658 (1.9)	14 (0.2)	3,036 (82.9)	5,478 (43.5)	2,959 (19)	255 (3.9)
Household using clean cooking fuel^&^, *n* (%)	10,598 (9.4)	87 (0.8)	29 (0.4)	1,121 (8.8)	2,905 (8.3)	36 (0.4)	3,209 (87.5)	1,591 (12.6)	1,309 (8.4)	311 (4.7)
Birth by cesarean section	6,329 (5.7)	1,024 (9.1)	71 (1.1)	1,418 (11.3)	1,045 (3.0)	86 (1.3)	412 (11.4)	1,619 (13.2)	560 (3.4)	94 (1.5)

*Denominator includes women who completed a pregnancy before enrolment.

†Among those who had previous livebirth(s).

‡Includes piped water, public tab, tube well, or rain water collection.

#Includes flush/pour flush toilet.

& Includes electricity, liquid petroleum gas, or kerosene.

Women were mainly from rural, resource-limited environments. On average, women were 25 years old, 67% had attended school, and 71% had a previous livebirth. Approximately 9% were adolescent mothers aged 15 to 19 years, and the proportion was high in 3 sites in sub-Saharan Africa, Zambia (24.5%), Kenya (19.0%), and DRC (17.3%) and was lowest in India Shivgarh site (1.1%). One-fifth of women had a previous miscarriage (previous pregnancies ended before 6 months), 9% had a previous stillbirth, and 3.3% had previous preterm birth (previous pregnancies end more than 1 month before time). Approximately 74% had access to an improved water source, 15% had access to toilet facility, and 9.4% lived in a household with clean cooking fuel. However, wide variations in these sociodemographic characteristics were observed by site and within the country (Table 1). For example, Karachi site was predominantly urban and peri-urban areas, while Matiari site was in rural areas.

### Direct maternal morbidity

Overall, 32.7% of the pregnancies had at least one morbidity; with double the burden of morbidities in South Asia compared to sub-Saharan Africa (44.0% South Asia; 23.8% in sub-Saharan Africa) (Table 2). Women had 2 or more morbidities. For example, 35% of women with obstructed labour had at least one other comorbidity; and 32% of women with hypertensive disorder had a comorbidity. Within the region, there were large variations in burden of morbidity, particularly in late antepartum infection (5.6% in Bangladesh to 26.6% in Matiari in South Asia) (Table A in S1 Table; S1–S8 Figs). Pooled data, which represent the average across sites in the region, should be interpreted along with the range of morbidity burden across sites.

**Table 2:** Burden of direct maternal morbidities.

	South Asia	sub-Saharan Africa	Overall
Pregnancies that lasted 28 weeks or more, or resulted in death, N^*^	**61,972**	**52,078**	**114,050**
**Obstetric haemorrhage** Antepartum haemorrhage; number, %, (95% CI)^†^			
	1,686, 3.1% (1.6% to 4.6%)	742, 1.5% (1% to 2%)	2,428, 2.2% (1.5% to 2.9%)
Severe postpartum haemorrhage; number, %, (95% CI)^‡^	1,268, 2.6% (1.5% to 3.7%)	458, 1% (0.7% to 1.3%)	1,726, 1.7% (1.2% to 2.2%)
**Hypertensive disorder of pregnancy**			
Hypertension only; number, %, (95% CI) ^#^	5,228, 8.2% (3.3% to 13.1%)	3,777, 6.7% (3% to 10.1%)	9,005, 7.4% (4.6% to 10.1%)
Preeclampsia or eclampsia; number, %, (95% CI)^#^	1,066, 1.8% (1.3% to 2.2%)	756, 1.2% (0.4% to 2%)	1,822, 1.4% (0.9% to 2%)
**Pregnancy-related infection**			
Late third trimester antepartum infection; number, %, (95% CI)^β^	8,295, 16.1% (8.4% to 23.8%)	1,875, 3.5% (1.4% to 5.7%)	10,170, 9.1% (5.6% to 12.6%)
Postpartum infection; number, %, (95% CI)^∞^	11,167, 16.3% (9.9% to 22.6%)	1,170, 2.4% (1.4% to 3.5%)	12,337, 8.6% (4.4% to 12.8%)
**Prolonged or obstructed labour;** number, %, (95% CI)^"^	12,636, 13.1% (0.6% to 25.6%)	4,607, 9.5% (6.8% to 12.3%)	17,243, 11.1% (5.4% to 16.8%)
**Any of the above morbidities;** number, %, (95% CI)^**€**^	29,451, 44.0% (27.9% to 60.0%)	11,758, 23.8% (19.8% to 27.7%)	41,209, 32.7% (22.2% to 43.3%)

*A total of 183 women lost to follow-up, 92 in South Asia and 91 in sub-Saharan Africa.

^†^Missing data for 101 deliveries in South Asia, 106 in sub-Saharan Africa.

^‡^Missing data for 796 deliveries in South Asia, 3,341 in sub-Saharan Africa.

^#^Missing data for 305 deliveries in South Asia, 1,689 in sub-Saharan Africa.

^β^Missing data for 828 deliveries in South Asia, 3,358 in sub-Saharan Africa.

^∞^Missing data for 795 deliveries in South Asia, 3,312 in sub-Saharan Africa.

^"^Missing data for 834 deliveries in South Asia, 3,332 in sub-Saharan Africa.

^€^Missing data for 1,478 deliveries in South Asia, 4,744 in sub-Saharan Africa.

About 2.2% (95% CI 1.5% to 2.9%) had antepartum haemorrhage, and 1.7% (95% CI 1.2% to 2.2%) had severe postpartum haemorrhage. Overall, 1.4% (95% CI 0.9% to 2.0%) women suffered from preeclampsia or eclampsia, and 7.4% (95% CI 4.6% to 10.1%) women had gestational hypertension alone. About 11.1% (95% CI 5.4% to 16.8%) women reported having prolonged or obstructed labour. Clinical features of late antepartum infection were present in 9.1% (95% CI 5.6% to 12.6%) and those of postpartum infection in 8.6% (95% CI 4.4% to 12.8%) women.

The burden of pregnancy-related infection in South Asia was substantially higher than that in sub-Saharan Africa (late antepartum infection 16.1% versus 3.5%, postpartum infection 16.3% versus 2.4%). The burden of obstetric haemorrhage was also higher in South Asia in comparison to that in sub-Saharan Africa (antepartum haemorrhage 3.1% versus 1.5%, and postpartum haemorrhage 2.6% versus 1.0%).

In contrast, the burden of hypertensive disorders of pregnancy was more similar in both South Asia and sub-Saharan Africa (hypertension only 8.2% versus 6.7%, preeclampsia or eclampsia 1.8% versus 1.2%). This was also the case with burden of prolonged or obstructed labour (13.1% versus 9.5%).

### Adverse pregnancy outcomes

Pregnancy-related deaths, neonatal mortality, and stillbirth rates were high in this population (Tables B and C in S1 Table) [22].

The pregnancy-related death rate was 187 per 100,000 births with the major share of those deaths happening around the time of labour and day of birth labour/delivery (65 per 100,000 births). We used total birth (all live and stillbirths) as the denominator.

Stillbirth rate was 27 deaths per 1,000 births with high regional variation (South Asia: 38; sub-Saharan Africa: 18). Thirteen per 1,000 births occurred in the antepartum period, and 9 per 1,000 births occurred in the intrapartum period. The timings of death in the remaining 6 deaths per 1,000 births were indeterminate, and verbal autopsy was not done.

The neonatal mortality rate in this cohort was 28 per 1,000 livebirths, again, with wide variation by region (South Asia: 41; sub-Saharan Africa: 18). A large proportion (44%) of newborn deaths occurred within the first 24 hours of life, and 30% occurred from day 1 to 6 after birth.

### Direct maternal morbidities and pregnancy-related deaths

In an analysis adjusted for baseline characteristics, severe postpartum haemorrhage (odds ratio (OR): 28.8, 95% CI 20.3 to 40.7), preeclampsia or eclampsia (OR: 9.13, 95% CI 6.10 to 13.7), and late antepartum maternal infection (OR: 2.80, 95% CI 1.63 to 4.80) increased the risk of pregnancy-related death. Other direct maternal morbidities were not correlated with pregnancy-related deaths (Table 3). Among socioeconomic and maternal characteristics, multiple births (a birth resulting in 2 or more children), previous history of C-section, wealth quintile, and older age (35 to 49 years of age) were most strongly associated with pregnancy-related death (Table D in S1 Table). We looked into the result by region, and these results were similar across regions.

**Table 3:** Association of maternal morbidities with pregnancy-related deaths.

Maternal morbidity	Pregnancy-related deaths per 100,000 births (95% CI)	Adjusted OR* (95% CI)	*p*-value
**Hypertensive disorder of pregnancy**^**†**^			
None	187 (162 to 216)	1	
Hypertension only	111 (60 to 207)	0.62 (0.33 to 1.19)	0.157
Preeclampsia or eclampsia	1,689 (1,190 to 2,392)	9.13 (6.1 to 13.7)	<0.001
**Antepartum haemorrhage**			
No	193 (169 to 221)	1	
Yes	699 (446 to 1,093)	2.80 (1.63 to 4.80)	<0.001
**Late antepartum maternal infection**^**‡**^			
No	174 (150 to 202)	1	
Yes	127 (74 to 220)	0.55 (0.29 to 1.01)	0.057
**Prolonged or obstructed labour**^**#**^			
No	175 (150 to 204)	1	
Yes	158 (109 to 231)	0.77 (0.50 to 1.21)	0.255
**Severe postpartum haemorrhage**^**β**^			
No	118 (100 to 141)	1	
Yes	3,240 (2,507 to 4,179)	28.8 (20.3 to 40.7)	<0.001
**Postpartum maternal infection**^**∞**^			
No	150 (128 to 177)	1	
Yes	322 (237 to 439)	1.83 (1.245 to 2.69)	0.002

*.Multivariable models included woman’s age, woman’s school attendance, previous livebirth, multiple births, wealth quintile, and site.

^†^Missing data for 3 outcomes out of 232 pregnancy-related deaths (1.3%).

^‡^Missing data for 47 outcomes (20.3%).

^#^Missing data for 45 outcomes (19.4%).

^β^Missing data for 48 outcomes (20.7%).

^∞^Missing data for 47 outcomes (20.3%).

### Direct maternal morbidities and stillbirths

In a multivariate analysis, all measured direct maternal morbidities were associated with antepartum stillbirths. The strongest associations with antepartum stillbirths were with preeclampsia or eclampsia (OR: 3.71, 2.99 to 4.61), severe postpartum haemorrhage (OR: 3.7, 2.98 to 4.60), and antepartum haemorrhage (OR: 3.57, 2.96 to 4.32) (Table 4). Of socioeconomic and woman’s characteristics, multiple births, previous history of stillbirth and preterm birth, and mother’s age (35 to 49 years old) were strongly correlated with antepartum stillbirths (Table E in S1 Table).

**Table 4:** Adjusted association of maternal morbidities with ASBs and ISBs.

	ASBs			ISBs		
	ASB per 1,000 births (95% CI)	Adjusted OR* (95% CI)	*p*-value	ISB per 1,000 births (95% CI)	Adjusted OR* (95% CI)	*p*-value
**Maternal morbidity**						
**Hypertensive disorder of pregnancy**^**†**^	3					
None	14 (14 to 15)	1		11 (10 to 11)	1	
Hypertension only	25 (21 to 28)	1.40 (1.20 to 1.63)	<0.001	11 (9 to 14)	1.01 (0.82 to 1.26)	0.885
Preeclampsia/eclampsia	61 (51 to 74)	3.71 (2.99 to 4.61)	<0.001	17 (12 to 25)	1.46 (0.99 to 2.15)	0.058
**Antepartum haemorrhage**						
No	15 (14 to 16)	1		10 (10 to 11)	1	
Yes	60 (51 to 70)	3.57 (2.96 to 4.32)	<0.001	33 (27 to 42)	2.86 (2.23 to 3.66)	<0.001
**Late antepartum maternal infection**^**‡**^						
No	15 (14 to 16)	1		10 (10 to 11)	1	
Yes	29 (25 to 32)	1.58 (1.37 to 1.82)	<0.001	17 (15 to 20)	1.33 (1.11 to 1.59)	0.002
**Prolonged or obstructed labour**^**#**^						
No	15 (14 to 16)	1		9 (9 to 10)	1	
Yes	23 (20 to 25)	1.46 (1.29 to 1.67)	<0.001	19 (17 to 22)	1.87 (1.62 to 2.16)	<0.001
**Severe postpartum haemorrhage**^**β**^						
No	15 (14 to 16)	1		10 (10 to 11)	1	
Yes	62 (51 to 75)	3.7 (2.98 to 4.60)	<0.001	31 (23 to 40)	2.66 (1.98 to 3.56)	<0.001
**Postpartum maternal infection**^**∞**^						
No	14 (14 to 15)	1		10 (9 to 10)	1	
Yes	29 (26 to 32)	1.70 (1.50 to 1.93)	<0.001	19 (17 to 22)	1.57 (1.35 to 1.83)	<0.001

*Multivariable models included, woman's age, woman's school attendance, previous livebirth, multiple births, wealth quintile and site.

^†^ Missing data for 16 ASB and 10 ISB

^‡^ Missing data for 52 ASB and 38 ISB.

^#^Missing data for 50 ASB and 41 ISB.

^β^ Missing data for 55 ASB and 43 ISB

^∞^ Missing data for 49 ASB and 37 ISB.

ASB, antepartum stillbirth; ISB, intrapartum stillbirths; OR, odds ratio.

Intrapartum stillbirths were most strongly associated with antepartum haemorrhage (OR: 2.86, 2.23 to 3.66), severe postpartum haemorrhage (OR: 2.66, 1.98 to 3.56), and prolonged or obstructed labour (OR: 1.87, 1.62 to 2.16) (Table 4). Of socioeconomic and woman’s characteristics, multiple births, previous stillbirth, first pregnancy, and wealth quintile were strongly associated with increased risk for intrapartum stillbirths (Table E in S1 Table).

### Direct maternal morbidities and neonatal deaths

In a multivariate analysis, all measured direct maternal morbidities were associated with neonatal deaths. The morbidities strongly associated with neonatal death were antepartum haemorrhage (OR: 2.21, 95% CI: 1.87 to 2.63), severe postpartum haemorrhage (OR: 1.89, 1.53 to 2.33), and preeclampsia or eclampsia (OR: 1.76, 95% CI: 1.41 to 2.20) (Table 5). Socioeconomic and maternal characteristics strongly associated with increased risk of neonatal deaths were multiple births, previous history of stillbirth, first pregnancy, wealth quintile, and previous history of preterm birth (Table F in S1 Table).

**Table 5:** Adjusted association of maternal morbidities with NNDs.

	NNDs		
Maternal morbidity	NND	Adjusted OR*	*p*-value
	per 1,000 livebirth	(95% CI)	
	(95% CI)		
**Hypertensive disorder**^**†**^			
None	30 (29 to 31)	1	
Hypertension only	34 (31 to 38)	1.07 (0.94 to 1.22)	0.295
Preeclampsia/eclampsia	57 (47 to 69)	1.76 (1.41 to 2.20)	<0.001
**Antepartum haemorrhage**			
No	29 (28 to 30)	1	
Yes	73 (63 to 85)	2.21 (1.87 to 2.63)	<0.001
**Late antepartum maternal infection**^**‡**^			
No	27 (26 to 29)	1	
Yes	53 (48 to 57)	1.52 (1.38 to 1.69)	<0.001
**Prolonged or obstructed labour**^**#**^			
No	27 (26 to 28)	1	
Yes	44 (41 to 47)	1.49 (0.35 to 1.63)	<0.001
**Severe postpartum haemorrhage**^**β**^			
No	29 (28 to 30)	1	
Yes	67 (56 to 81)	1.89 (1.53 to 2.33)	<0.001
**Postpartum maternal infection**^**∞**^			
No	27 (26 to 28)	1	
Yes	55 (51 to 59)	1.63 (1.48 to 1.79)	<0.001

*Multivariable models included, woman’s age, woman’s school attendance, previous livebirth, multiple births, wealth quintile, and site.

^†^Missing data for 36 out of 3,343 NND (1.1%).

^‡^Missing data for 125 outcomes (3.7%).

^#^Missing data for 129 outcomes (3.9%).

^β^Missing data for 120 outcomes (3.6%).

^∞^Missing data for 120 outcomes (3.6%).

NND, neonatal death; OR, odds ratio.

## Discussion

This large prospective cohort study conducted in about 114,000 pregnant women in South Asia and sub-Saharan Africa shows that a third of women suffer from a direct maternal morbidity. The proportion of women with direct maternal morbidity was greater in South Asia (44%) than in sub-Saharan Africa (24%), largely because of a greater prevalence of pregnancy-related infections. Pregnancy-related death, neonatal mortality, and stillbirth rates were similar to the study based on larger AMANHI mortality study [22]. The study clearly demonstrated that direct maternal morbidity is associated with pregnancy-related deaths, stillbirths, and neonatal deaths.

A review of available systematic reviews reported 27 million morbid episodes from 5 main direct obstetric causes, among 210 million pregnancies (13%) globally in 2015 [23]. Another major previous source of maternal morbidity data was the WHO multicountry survey, which provided data from a third of a million women who attended district or tertiary hospitals in 29 LMICs [24]. Most of the previous data are from hospital-based studies, and studies have lacked common definitions and standard identification criteria [25]. The major strengths of our study are its prospective, population-based, cohort design, large sample size, and harmonised data collection schedule and tools, training, implementation, and common definitions of maternal morbidity at all the study sites. We identified all pregnant women in the study population using 1 to 3 monthly reproductive surveillance sweeps and prospectively followed up 95% of all reported pregnancies to the end of the postpartum period; thus, possibility of recall and reporting bias was minimised. Our study was conducted in South Asia and sub-Saharan Africa, the 2 regions with the highest burden of maternal and newborn mortality and morbidity and with the greatest paucity of data.

We included pregnancies that lasted at least for 28 weeks (or those of shorter duration that resulted in death of the pregnant woman or a live-born neonate) in the study because of the following reasons. Maternal morbidity information was collected for the first time at 24 to 28 weeks because most women present quite late during pregnancy. However, if a pregnant woman died at any gestation, or a live-born neonate with <28 weeks gestation died, information on morbidity was collected by a verbal autopsy. We felt that it was not appropriate to present morbidity information for the remaining pregnancies that ended <28 weeks (considered to be miscarriages or abortions based on WHO definition) because our study was likely to miss most of these cases because of late reporting of pregnancy and would thus be a serious underreporting.

Our study has some limitations. Even though the sample size was large, population of a geographically limited site per country was included, and it is unlikely that the study sites are representative of the entire country and region. Second, morbidity information was collected only at 5 contacts during pregnancy and postpartum period, and not all enrolled women had all these contacts, which could have resulted in some underestimation of morbidity. Third, we did not collect information on indirect maternal morbidities such as anaemia or gestational diabetes. Fourth, as we only presented data on infections in the late antenatal period, we are likely to have underestimated the total burden of pregnancy-related infections, and it was not possible to examine the aetiology of infections using these self-reported data. Fifth, although all the visits during pregnancy and after birth were all conducted at home/in the community in Zambia site, there may be possibility that the different enrolment strategy in Zambia may have led to an underestimate of pregnancy-related deaths, stillbirths, and neonatal deaths in that site. Finally, except hypertensive disorders of pregnancy for which blood pressure was measured and urine examination done at each visit, all other morbidities were based on self-reporting of symptoms and signs by the pregnant women.

This design proposed by the reviewer is appropriate for settings with well-established health systems that are universally accessible, but any such validation is likely to be incomplete in settings where most women do not seek care for their morbidity and biased towards an identification of the severest morbidities only. We were seeking to derive community-based estimates of morbidity in this study. In addition, even in a setting where clinical records are accessible, the information that could be retrieved is likely to be poor quality. Therefore, while we did consider collecting facility-based information in the study for triangulation purposes, this was not found to be feasible. Instead, we focused on improving the quality of the data that we could collect in the community, using periodic observation of blood pressure and proteinuria for our preeclampsia estimations for example. However, other morbidities (e.g., haemorrhage, infections, and obstructed labour) were not amenable to such measurements, and, therefore, self-reporting was deemed to be most appropriate strategy for these settings. There is a possibility that differential understanding of questions could have contributed to variations in morbidity estimates across countries and regions, in addition to the true epidemiologic differences. However, rigorous training of field workers and the monthly random spot checks limit the scope of such potential bias.

We found a similar incidence of hypertensive disorders of pregnancy in South Asia (8.2%) and sub-Saharan Africa (6.7%). Most of these women had gestational hypertension, while 1.4% had preeclampsia or eclampsia (1.8% in South Asia and 1.2% in sub-Saharan Africa). Previous study and a review have reported that a higher proportion of pregnant women have preeclampsia (2.3%) and eclampsia (0.5%) [23,24]. This difference may be explained by population-based nature of our cohort, because hospital-based data tend to have a higher prevalence of severe morbidity. Incidence of hypertensive disorders of pregnancy in our study was similar to the published rate of 6% to 8% in a community-based study in less-developed settings [26]. The same study, however, reported higher incidence of preeclampsia or eclampsia (2% to 4%). This rate was higher than ours by 2-fold. This could be because our definition of preeclampsia was based on hypertension and presence of proteinuria only, whereas Magee’s definition was gestational hypertension plus proteinuria or a preeclampsia-defining signs and symptoms (headache, visual symptoms, chest pain, etc.).

Two previous systematic reviews reported that 1.7% and 2.8% women had severe postpartum haemorrhage, respectively [27,28]. The corresponding proportion in our study was 1.7% (2.6% in South Asia and 1.0% in sub-Saharan Africa). Thus, our findings are similar despite using a different definition of severe postpartum haemorrhage (haemorrhage accompanied by loss of consciousness, or the need for blood transfusion or surgery) rather than the previously used 1,000 ml blood loss. Research indicated that women are unable to accurately report on the amount of blood loss. We also found that about 2.2% pregnant women (3.1% in South Asia and 1.5% in sub-Saharan Africa) have antepartum haemorrhage, but we could not find any systematic reviews or global estimates for this morbidity. We found one systematic review on placenta previa showing main conditions responsible for antepartum haemorrhage, which is consistent with our findings. This review showed that the prevalence was 1.2% of pregnancies in Asia and 0.3% of pregnancies in sub-Saharan Africa [29].

We observed that a large proportion of women had clinically suspected pregnancy-related infection, both in late third trimester (16.1% in South Asia and 3.5% in sub-Saharan Africa) as well as in the postpartum period (16.3% in South Asia and 2.4% in sub-Saharan Africa). A published systematic review showed pooled maternal infection estimates of approximately 4% in labour and about 3.5% in postpartum period [30]. The overwhelming majority of studies included in this review were from high-income country settings, and the diagnosis was made at health facilities. Another review based on review of hospital and community studies showed that global estimate of postpartum sepsis was 4.4% [31]. The higher rate of antepartum and postpartum infection in South Asia observed in our study may be related to higher risk of infections in high-density population settings. However, we used self-reported fever and vaginal discharge as a basis of ascertaining infections and therefore cannot rule out the possibility of overreporting by women in South Asia. Our aim was to derive community-based estimates of maternal infections from women’s self-reporting. However, large intraregion variations in the burden of late antepartum infections and postpartum infections particularly in South Asia could imply that self-reported infections signs were challenging to collect. Overcrowding, poorer hygiene, lower education, and higher rates of malnutrition in South Asian sites could also be potential explanatory factors. The high burden of pregnancy-related infection may be an important risk factor for stillbirth, preterm birth, and early neonatal sepsis. The remarkable difference in infection rates between Asia and Africa should be explored further in future studies.

In this study, 11.1% women reported prolonged or obstructed labour, 13.1% in South Asia, and 9.5% in sub-Saharan Africa. These proportions, particularly in South Asia, were somewhat higher than those reported (8.7%) [32–33] A high proportion of home deliveries and poor quality of childbirth care in facilities could have contributed to the high rates that we observed. It may also be due to difficulty of women knowing exactly when labour started.

The intraregional and interregional variations in the burden of direct maternal morbidity in our study could be because of epidemiological and health system contexts of different sites. Overcrowding, sanitation, literacy, and fertility were different across sites (Table 1) Quality of care during antepartum, intrapartum, and postpartum periods was also likely to quite different across study sites. A number of sociodemographic and health variables were significant risk factors for pregnancy-related deaths, stillbirths, and neonatal deaths (Tables C–E in S1 Table).

Relatively higher proportion of missing data was observed in pregnancy-related deaths than in stillbirths or in neonatal deaths. This is primarily because it is a rare outcome, and it was not possible to obtain any data on maternal morbidities if verbal autopsies were not conducted.

This study has important implications for public health programmes that aim to improve maternal, foetal, and newborn health and survival. First, the high burden of morbidity and clear evidence of its association with pregnancy-related deaths, stillbirth, and neonatal deaths highlight the need for improving health of women and mothers. This includes promotion of preconception health and high-quality antepartum, intrapartum, and postpartum care. Second, antenatal care models with a minimum of 8 contacts recommended by WHO will help in early identification and treatment of morbidity. In addition to improving the health system, there is a need to improve community-based interventions to prevent, identify, and refer pregnant women with morbidities to help address ongoing challenges in appropriate care for these women. Second, the quantitative estimates of disease burden can be used to more efficiently plan health services. This implies having adequate infrastructure, strong referral network, health worker, and medical supplies to treat common morbidities. Finally, given the limitations of the current global and regional estimates of maternal morbidity, it is important that our estimates from population-based prospective cohorts are used to improve them. We believe that implementation of effective strategies for prevention and management of maternal morbidity will help women, foetuses, and newborns survive and thrive and accelerate progress towards achieving Sustainable Development Goals.

## Supporting information

S1 Fig Burden of hypertensive disorder of pregnancy (hypertension only) by site. (TIF)

S2 Fig Burden of hypertensive disorder of pregnancy (preeclampsia or eclampsia) by site. (TIF)

S3 Fig Burden of antepartum haemorrhage by site. (TIF)

S4 Fig Burden of late antepartum maternal infection by site. (TIF)

S5 Fig Burden of prolonged/obstructed labour by site. (TIF)

S6 Fig Burden of severe postpartum haemorrhage. (TIF)

S7 Fig Burden of postpartum maternal infection. (TIF)

S8 Fig Burden of any morbidity. (TIF)

S1 Table Supporting information tables. Table A. Burden of maternal morbidities by site. Table B. Burden of pregnancy-related deaths, stillbirths, and neonatal deaths. Table C. Burden of pregnancy-related deaths, stillbirths, and neonatal deaths by site. Table D. Adjusted association of background characteristics and maternal morbidities with pregnancy-related death (PRD). Table E. Adjusted association of background characteristics with antepartum stillbirths (ASBs) and intrapartum stillbirths (ISBs). Table F. Adjusted association of background characteristics with neonatal deaths (NNDs).
(DOCX)

S1 Text Study protocol and core variable tables. (DOCX)

## Abbreviations

AMANHI: Alliance for Maternal and Newborn Health Improvement
LMICs: low- and middle-income countries
OR: odds ratio
