# Supplementary figures and images for: Direct maternal morbidity and the risk of pregnancy-related deaths, stillbirths, and neonatal deaths in South Asia and sub-Saharan Africa: A population-based prospective cohort study in 8 countries
Source: PLoS Med. 2021 Jun 28;18(6):e1003644. doi: 10.1371/journal.pmed.1003644 (PMC8277068; doi:10.1371/journal.pmed.1003644)

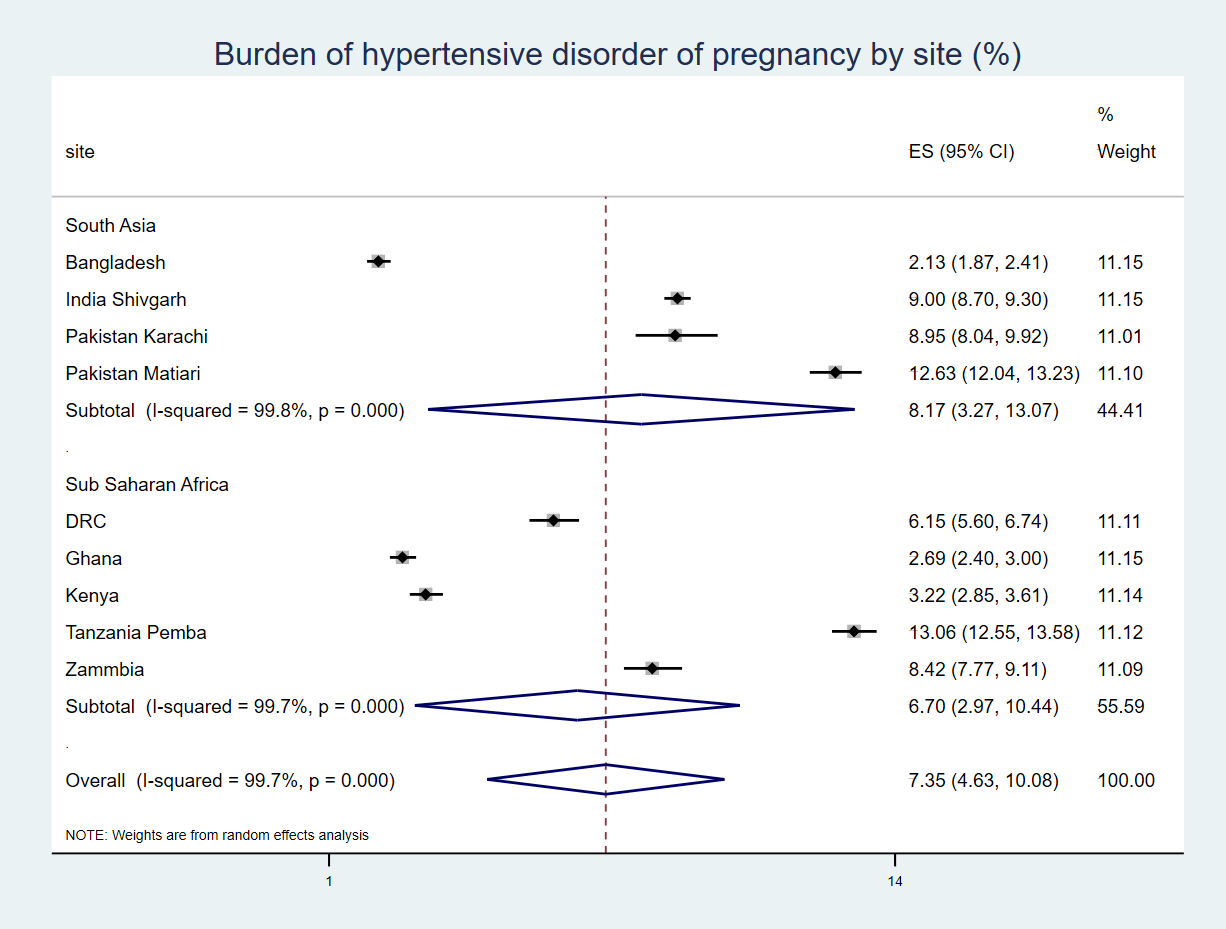

Supplement: S1 Fig — (TIF) [file pmed.1003644.s001.tif]

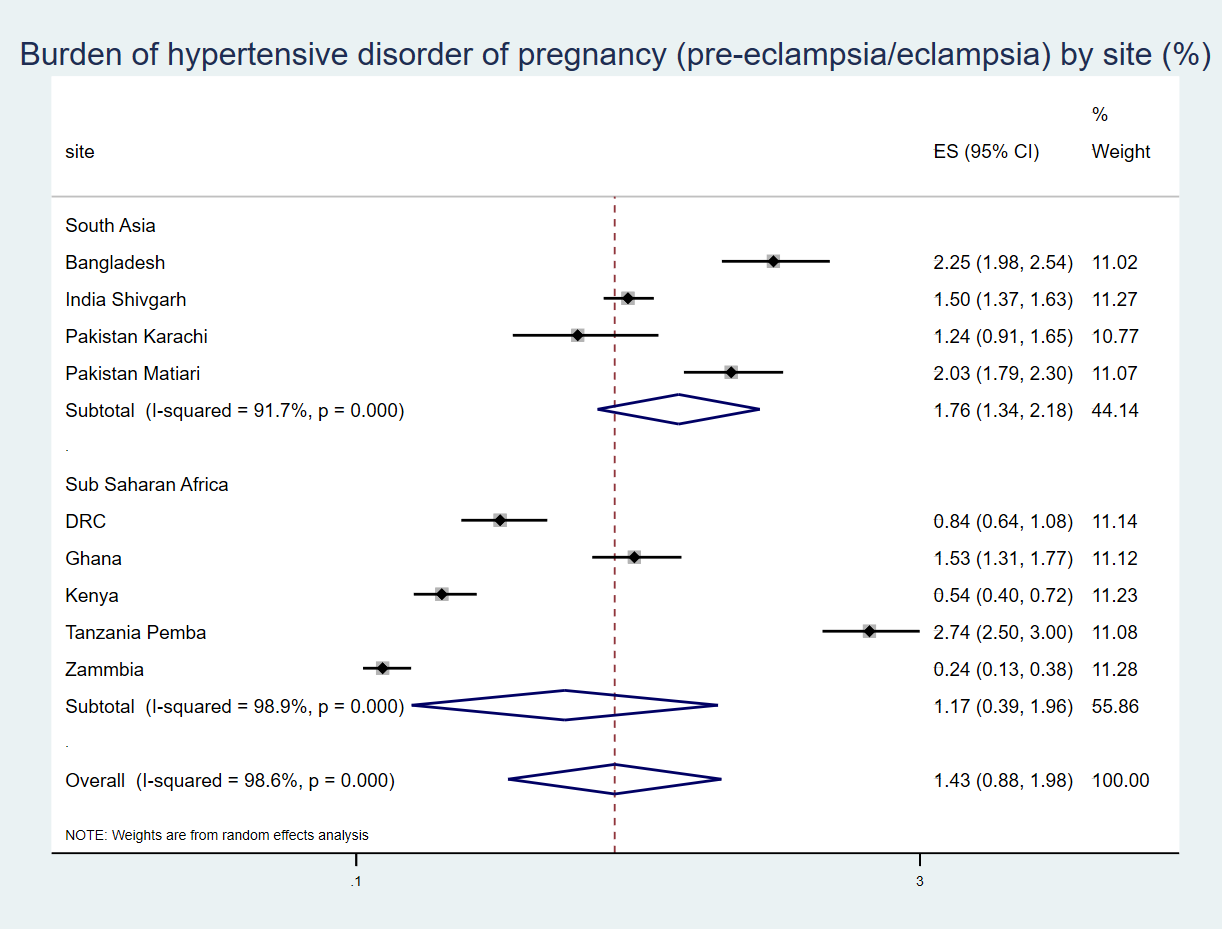

Supplement: S2 Fig — (TIF) [file pmed.1003644.s002.tif]

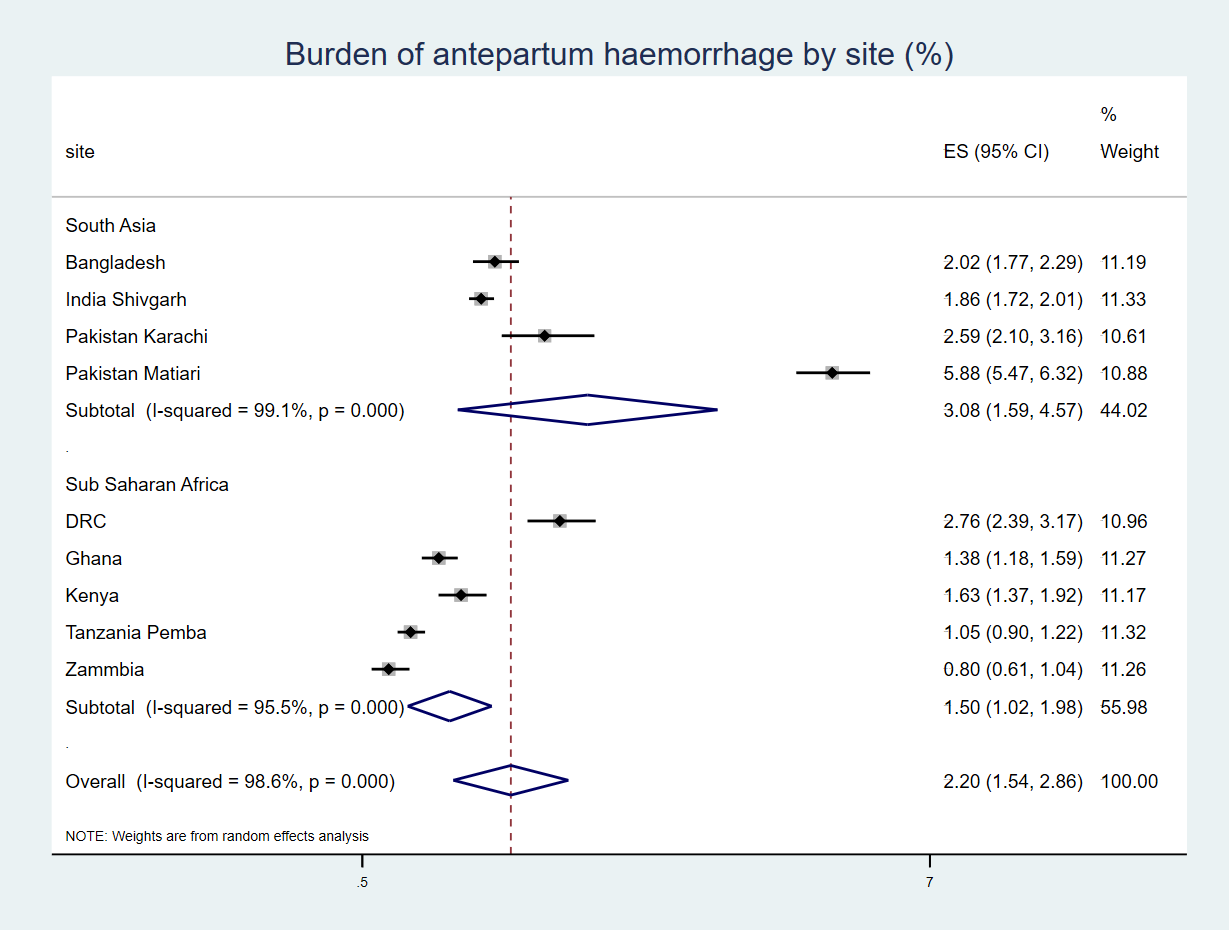

Supplement: S3 Fig — (TIF) [file pmed.1003644.s003.tif]

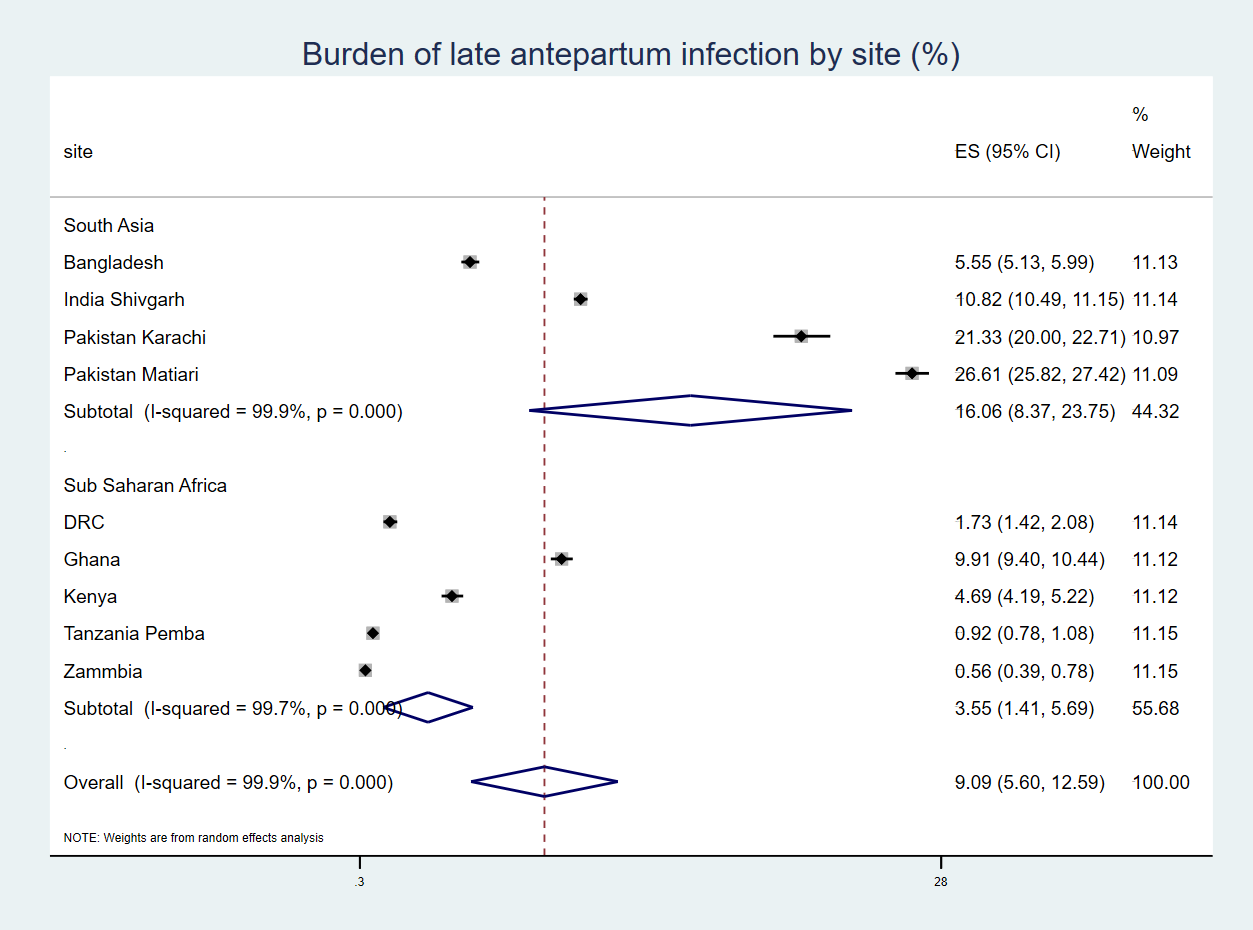

Supplement: S4 Fig — (TIF) [file pmed.1003644.s004.tif]

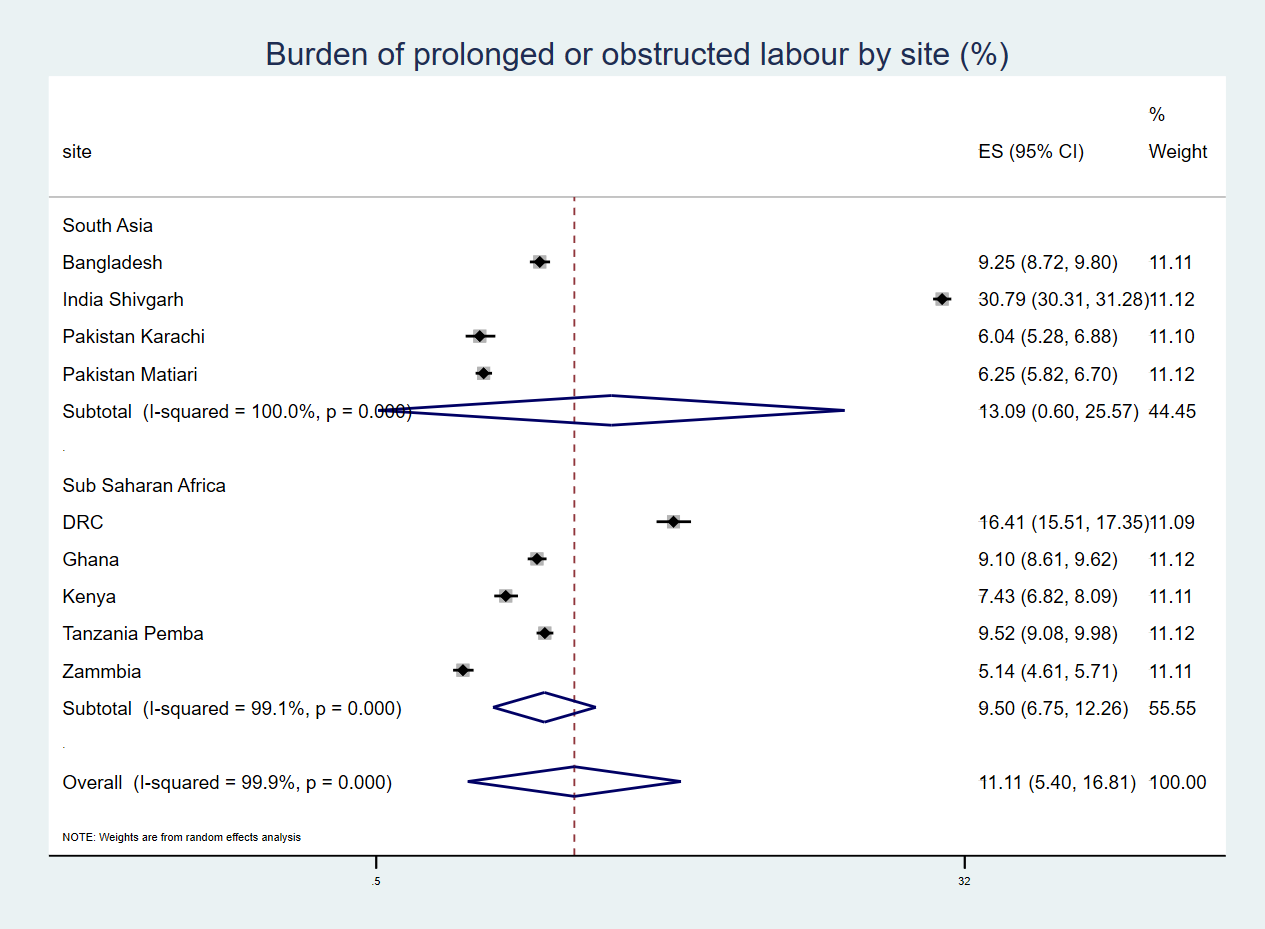

Supplement: S5 Fig — (TIF) [file pmed.1003644.s005.tif]

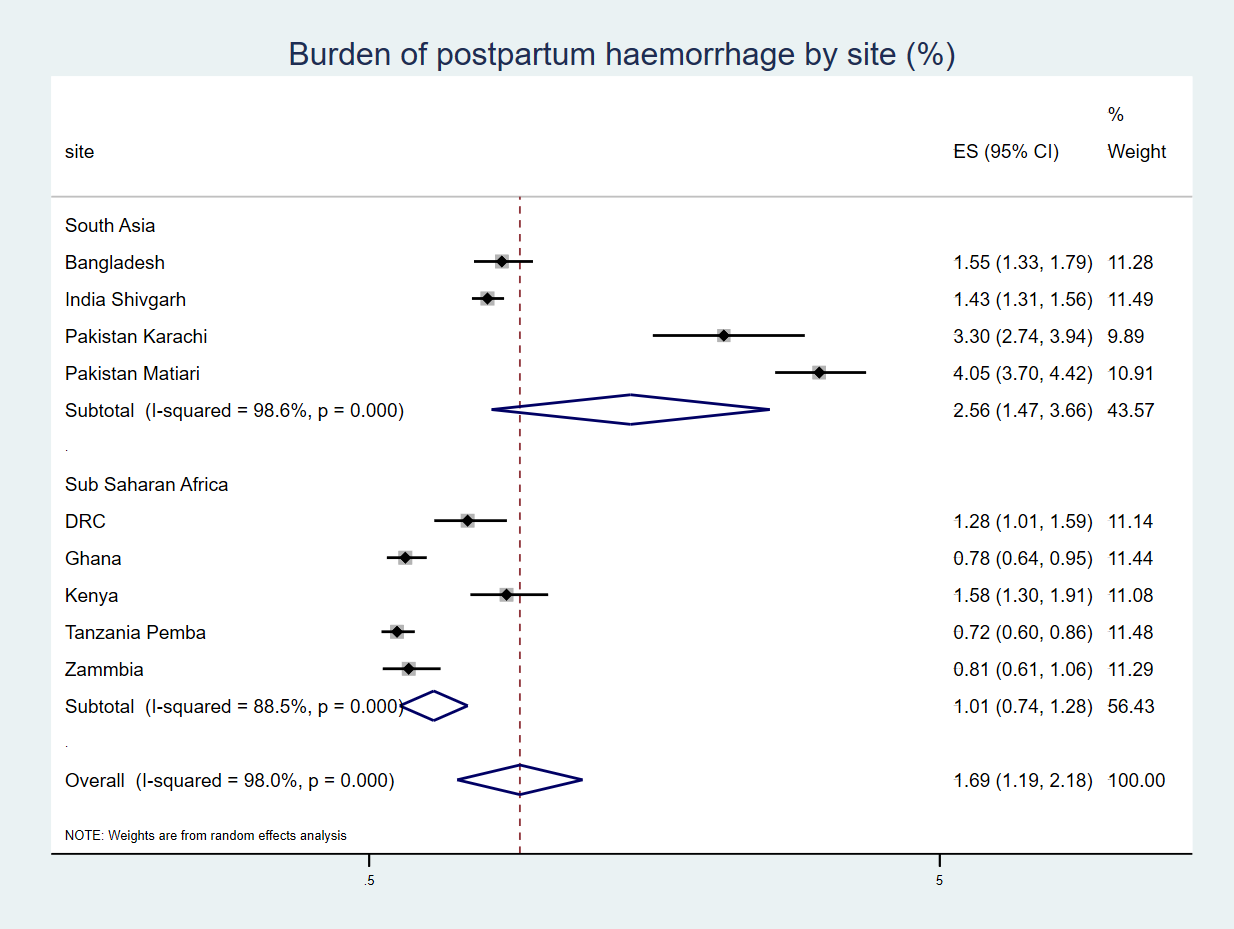

Supplement: S6 Fig — (TIF) [file pmed.1003644.s006.tif]

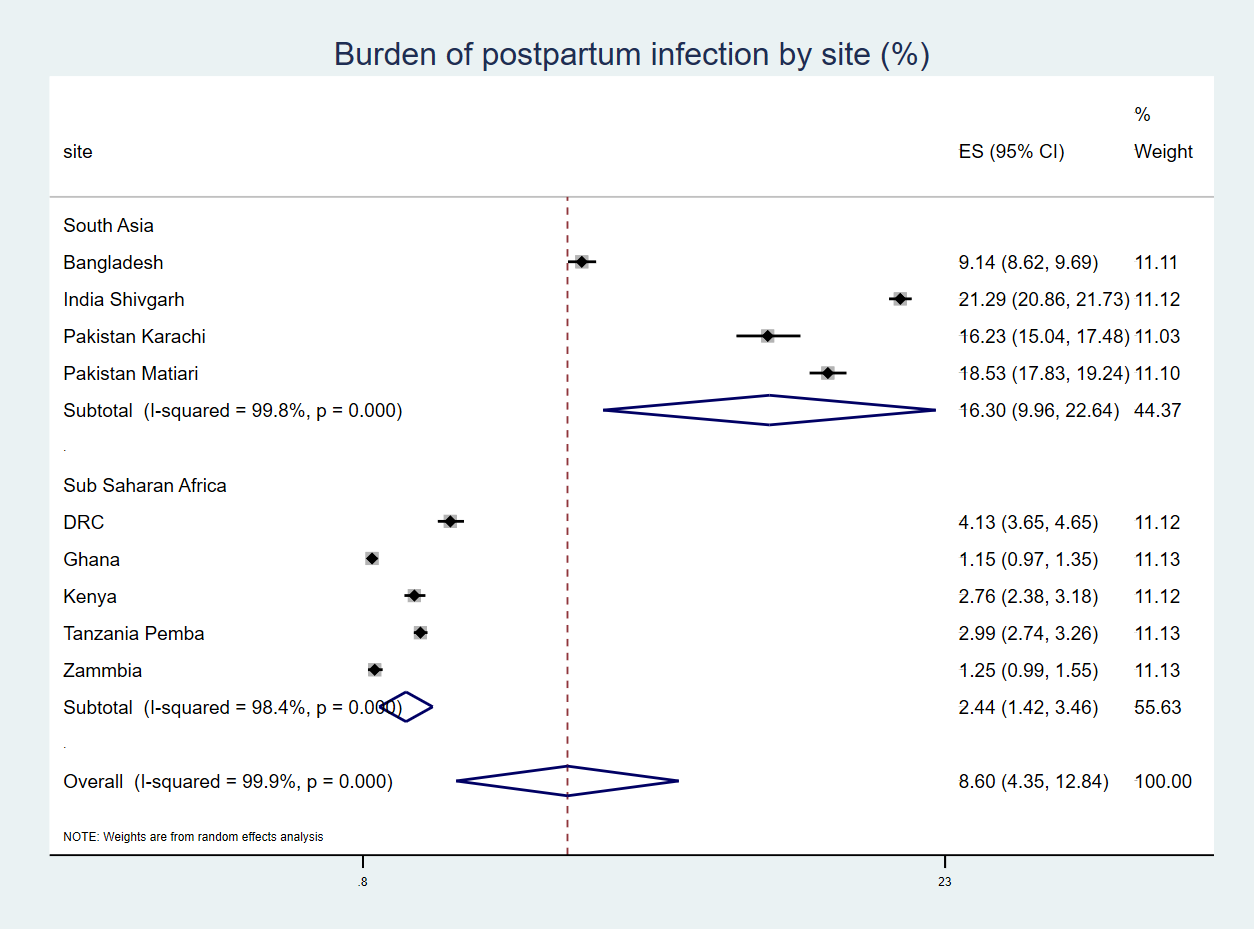

Supplement: S7 Fig — (TIF) [file pmed.1003644.s007.tif]

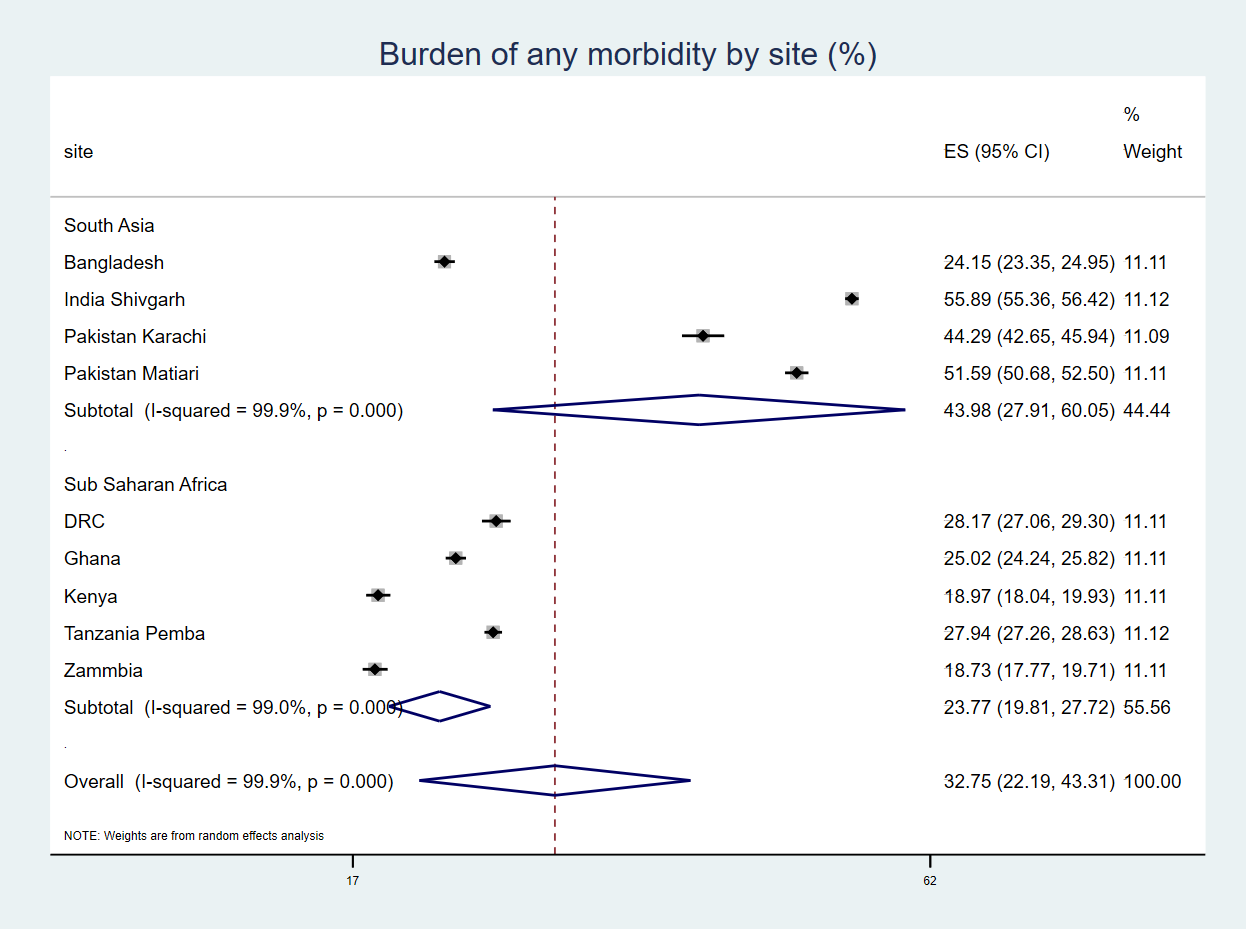

Supplement: S8 Fig — (TIF) [file pmed.1003644.s008.tif]
