# Supplementary material for: Direct maternal morbidity and the risk of pregnancy-related deaths, stillbirths, and neonatal deaths in South Asia and sub-Saharan Africa: A population-based prospective cohort study in 8 countries
Source: PLoS Med. 2021 Jun 28;18(6):e1003644. doi: 10.1371/journal.pmed.1003644 (PMC8277068; doi:10.1371/journal.pmed.1003644)
Supplement: S1 Table — Table A. Burden of maternal morbidities by site. Table B. Burden of pregnancy-related deaths, stillbirths, and neonatal deaths. Table C. Burden of pregnancy-related deaths, stillbirths, and neonatal deaths by site. Table D. Adjusted association of background characteristics and maternal morbidities with pregnancy-related death (PRD). Table E. Adjusted association of background characteristics with antepartum stillbirths (ASBs) and intrapartum stillbirths (ISBs). Table F. Adjusted association of background characteristics with neonatal deaths (NNDs). (DOCX) [file pmed.1003644.s009.docx]

# Supplementary tables

**Table A in S1 Table: Burden of maternal morbidities by site**

**Table B in S1 Table: Burden of pregnancy related deaths, stillbirths and neonatal deaths**

**Table C in S1 Table: Burden of pregnancy related deaths, stillbirths and neonatal deaths by site**

**Table D in S1 Table: Adjusted association of background characteristics with pregnancy related death (PRD)**

**Table E in S1 Table: Adjusted association of background characteristics with antepartum stillbirths (ASB) and intrapartum stillbirths (ISB)**

**Table F in S1 Table: Adjusted association of background characteristics with neonatal deaths (NND)**

| **Table A in S1 Table: Burden of maternal morbidities by site** | | | | | | |
| --- | --- | --- | --- | --- | --- | --- |
|  | **Bangladesh** | **DRC** | **Ghana** | **India-UP** | **Kenya** | **Pakistan Karachi** |
| Pregnancies which lasted 28 weeks or more, or resulted in death , **N ^1^** | 11309 | 6953 | 12810 | 34909 | 8521 | 3629 |
| **Obstetric haemorrhage** |  |  |  |  |  |  |
| Antepartum haemorrhage; number, % (95% CI) ^2^ | 227, 2 (1.8 to 2.3) | 194, 2.8 (2.4 to 3.2) | 176, 1.4 (1.2 to 1.6) | 651, 1.9 (1.7 to 2) | 139, 1.6 (1.4 to 1.9) | 94, 2.6 (2.1 to 3.2) |
| Severe postpartum haemorrhage; number, % (95% CI) ^3^ | 174, 1.5 (1.3 to 1.8) | 80, 1.3 (1 to 1.6) | 100, 0.8 (0.6 to 1) | 495, 1.4 (1.3 to 1.6) | 106, 1.6 (1.3 to 1.9) | 118, 3.3 (2.8 to 3.9) |
| **Hypertensive disorder of pregnancy** ^4^ |  |  |  |  |  |  |
| Hypertension only; number, % (95% CI) | 241, 2.2 (1.9 to 2.4) | 432, 6.2 (5.6 to 6.7) | 307, 2.7 (2.4 to 3) | 3131, 9 (8.7 to 9.3) | 274, 3.2 (2.9 to 3.6) | 325, 8.9 (8.1 to 9.9) |
| Pre to eclampsia/ Eclampsia; number, % (95% CI) | 254, 2.2 (2 to 2.5) | 59, 0.8 (0.7 to 1.1) | 175, 1.5 (1.3 to 1.8) | 521, 1.5 (1.4 to 1.6) | 46, 0.6 (0.4 to 0.7) | 45, 1.2 (0.9 to 1.7) |
| **Pregnancy related infection** |  |  |  |  |  |  |
| Late third trimester antepartum infection; number, %, (95%CI) ^5^ | 625, 5.5 (5.1 to 6) | 110, 1.7 (1.4 to 2.1) | 1261, 9.9 (9.4 to 10.4) | 3743 10.8 (10.5 to 11.1) | 314, 4.7 (4.2 to 5.2) | 764, 21.3 (20 to 22.7) |
| Postpartum maternal infection; number, %, (95%CI) ^6^ | 1029, 9.1 (8.6 to 9.7) | 260, 4.1 (3.7 to 4.7) | 146, 1.1 (1 to 1.3) | 7357, 21.3 (20.9 to 21.7) | 185, 2.8 (2.4 to 3.2) | 581, 16.2 (15.1 to 17.5) |
|  |  |  |  |  |  |  |
| **Prolonged or obstructed labour; number, %, (95%CI)** ^7^ | 1040, 9.2 (8.7 to 9.8) | 1033, 16.4 (15.5 to 17.3) | 1159, 9.1 (8.6 to 9.6) | 10639, 30.8 (30.3 to 31.3) | 498, 7.4 (6.8 to 8.1) | 216, 6 (5.3 to 6.9) |
|  |  |  |  |  |  |  |
| **Any of the above morbidities number, %, (95%CI)** ^8^ | 2688, 24.1 (23.3 to 24.9) | 1767, 28.2 (27.0 to 29.3) | 2912, 25.0 (21.2 to 25.8) | 19194, 55.9 (55.4 to 56.4) | 1274, 19.0 (18.0 to 19.9) | 1571, 44.3 (42.6 to 45.9) |
| ^1^ Missing data: Bangladesh (n=8) DRC (n=70) Ghana (n=14) India-UP (n=70) Kenya (n=1) Pakistan Karachi (n=3) Pakistan Matiari (n=14) Tanzania Pemba (n=5) Zambia (n=1). | | | | | | |
| 2 Missing data: Bangladesh (n=58) DRC (n=78) Ghana (n=55) India-UP (n=142) Kenya (n=7) Pakistan Karachi (n=57) Pakistan Matiari (n=468) Tanzania Pemba (n=43) Zambia (n=2). | | | | | | |
| 3 Missing data: Bangladesh (n=117) DRC (n=763) Ghana (n=94) India-UP (n=502) Kenya (n=1832) Pakistan Karachi (n=107) Pakistan Matiari (n=714) Tanzania Pemba (n=175) Zambia (n=578). | | | | | | |
| 4 Missing data: Bangladesh (n=62) DRC (n=90) Ghana (n=1416) India-UP (n=328) Kenya (n=14) Pakistan Karachi (n=57) Pakistan Matiari (n=482) Tanzania Pemba (n=70) Zambia (n=194) | | | | | | |
| 5 Missing data: Bangladesh (n=125) DRC (n=738) Ghana (n=124) India-UP (n=518) Kenya (n=1831) Pakistan Karachi (n=108) Pakistan Matiari (n=720) Tanzania Pemba (n=176) Zambia (n=590). | | | | | | |
| 6 Missing data: Bangladesh (n=117) DRC (n=737) Ghana (n=100) India-UP (n=495) Kenya (n=1826) Pakistan Karachi (n=107) Pakistan Matiari (n=719) Tanzania Pemba (n=175) Zambia (n=575). | | | | | | |
| 7 Missing data: Bangladesh (n=132) DRC (n=737) Ghana (n=101) India-UP (n=499) Kenya (n=1828) Pakistan Karachi (n=112) Pakistan Matiari (n=734) Tanzania Pemba (n=180) Zambia (n=585). | | | | | | |
| 8 Missing data: Bangladesh (n=228) DRC (n=825) Ghana (n=1207) India-UP (n=774) Kenya (n=1813) Pakistan Karachi (n=142) Pakistan Matiari (n=979) Tanzania Pemba (n=275) Zambia (n=726). | | | | | | |

| **S1 Table: Burden of maternal morbidities by site (continued)** | | |  |  | |
| --- | --- | --- | --- | --- | --- |
|  |  | **Pakistan Matiari** | **Tanzania Pemba** | **Zambia** | |
| **Pregnancies which lasted 28 weeks or more, or resulted in death, N ^1^** |  | 12125 | 16811 | 6983 | |
| **Obstetric haemorrhage** |  |  |  |  | |
| Antepartum haemorrhage; number, % (95% CI) ^2^ |  | 714, 5.9 (5.5 to 6.3) | 177, 1.0 (0.9 to 1.2) | 56, 0.8 (0.6 to 1) | |
| Severe postpartum haemorrhage; number, % (95% CI) ^3^ |  | 481, 4 (3.7 to 4.4) | 120, 0.7 (0.6 to 0.9) | 52, 0.8 (0.6 to 1.1) | |
| **Hypertensive disorder of pregnancy** ^4^ |  |  |  | |  |
| Hypertension only; number, % (95% CI) |  | 1531, 12.6 (12 to 13.2) | 2192, 13.1 (12.6 to 13.6) | 572, 8.4 (7.8 to 9.1) | |
| Pre to eclampsia/ Eclampsia; number, % (95% CI) |  | 246, 2 (1.8 to 2.3) | 460, 2.7 (2.5 to 3) | 16, 0.2 (0.1 to 0.4) | |
| **Pregnancy to related infection** |  |  |  |  | |
| Late third trimester antepartum infection; number, %, (95%CI) ^5^ |  | 3163, 26.6 (25.8 to 27.4) | 154, 0.9 (0.8 to 1.1) | 36, 0.6 (0.4 to 0.8) | |
| Postpartum maternal infection; number, %, (95%CI) ^6^ |  | 2200, 18.5 (17.8 to 19.2) | 499, 3 (2.7 to 3.3) | 80, 1.2 (1 to 1.6) | |
|  |  |  |  |  | |
| **Prolonged or obstructed labour; number, %, (95%CI)** ^7^ |  | 741, 6.2 (5.8 to 6.7) | 1588, 9.5 (9.1 to 10) | 329, 5.1 (4.6 to 5.7) | |
|  |  |  |  |  | |
| **Any of the above morbidities number, %, (95%CI)** ^8^ |  | 5998, 51.6 (50.7 to 52.5) | 4633, 27.9 (27.3 to 28.6) | 1172, 18.7 (17.8 to 19.7) | |
| ^1^ Missing data: Bangladesh (n=8) DRC (n=70) Ghana (n=14) India-UP (n=70) Kenya (n=1) Pakistan Karachi (n=3) Pakistan Matiari (n=14) Tanzania Pemba (n=5) Zambia (n=1). | | | | | |
| ^2^ Missing data: Bangladesh (n=58) DRC (n=78) Ghana (n=55) India-UP (n=142) Kenya (n=7) Pakistan Karachi (n=57) Pakistan Matiari (n=468) Tanzania Pemba (n=43) Zambia (n=2). | | | | | |
| ^3^ Missing data: Bangladesh (n=117) DRC (n=763) Ghana (n=94) India-UP (n=502) Kenya (n=1832) Pakistan Karachi (n=107) Pakistan Matiari (n=714) Tanzania Pemba (n=175) Zambia (n=578). | | | | | |
| ^4^ Missing data: Bangladesh (n=62) DRC (n=90) Ghana (n=1416) India-UP (n=328) Kenya (n=14) Pakistan Karachi (n=57) Pakistan Matiari (n=482) Tanzania Pemba (n=70) Zambia (n=194). | | | | | |
| ^5^ Missing data: Bangladesh (n=125) DRC (n=738) Ghana (n=124) India-UP (n=518) Kenya (n=1831) Pakistan Karachi (n=108) Pakistan Matiari (n=720) Tanzania Pemba (n=176) Zambia (n=590). | | | | | |
| ^6^ Missing data: Bangladesh (n=117) DRC (n=737) Ghana (n=100) India-UP (n=495) Kenya (n=1826) Pakistan Karachi (n=107) Pakistan Matiari (n=719) Tanzania Pemba (n=175) Zambia (n=575). | | | | | |
| ^7^ Missing data: Bangladesh (n=132) DRC (n=737) Ghana (n=101) India-UP (n=499) Kenya (n=1828) Pakistan Karachi (n=112) Pakistan Matiari (n=734) Tanzania Pemba (n=180) Zambia (n=585). | | | | | |
| ^8^ Missing data: Bangladesh (n=228) DRC (n=825) Ghana (n=1207) India-UP (n=774) Kenya (n=1813) Pakistan Karachi (n=142) Pakistan Matiari (n=979) Tanzania Pemba (n=275) Zambia (n=726). | | | | | |

| **Table B in S1 Table: Burden of pregnancy related deaths, stillbirths and neonatal deaths** | |  |  |
| --- | --- | --- | --- |
|  | **South Asia** | **Sub Saharan Africa** | **Overall** |
|  | 62,045 | 50,239 | 112,284 |
| **Pregnancies which lasted 28 weeks or more, or resulted in death with pregnancy related death outcome known** |  |  |  |
| **Pregnancy-related deaths***; number, ratio (95%CI) | 138, 222 (183 to 260) | 94, 172 (69 to 275) | 232, 187 (118 to 256) |
| Pregnancy-related deaths by time of death; number, ratio (95%CI) |  |  |  |
| 3rd trimester | 22, 24 (0 to 48) | 21, 18 (0 to 45) | 43, 20 (2 to 38) |
| Labour and day of birth | 67, 103 (76 to 130) | 24, 33 (4 to 62) | 91, 65 (32 to 98) |
| 2-7 days postpartum | 17, 26 (12 to 41) | 4, 7 (0 to 19) | 21, 14 (5 to 23) |
| 8-42 days postpartum | 22, 27 (3 to 50) | 14, 19 (2 to 36) | 36, 23 (10 to 36) |
| Timing unknown | 10, 11 (0 to 22) | 31, 39 (0 to 77) | 41, 18 (1 to 36) |
|  | 61,972 | 52,078 | 114,050 |
| **Pregnancies which lasted 28 weeks or more, or resulted in death with stillbirth outcome known** |  |  |  |
|  | 2401, 38 (32 to 46) | 1089, 18 (9 to 28) | 3490, 27 (18 to 37) |
| **Stillbirths****; number, rate (95%CI) |  |  |  |
|  |  |  |  |
| Stillbirths by time of death; number, rate (95%CI) |  |  |  |
| Antepartum stillbirth | 1156, 19 (11 to 26) | 470, 9 (3 to 16) | 1626, 13 (8 to 19) |
| Intrapartum stillbirth | 880, 13 (10 to 16) | 223, 5 (4 to 7) | 1103, 9 (6 to 12) |
| Timing unknown | 369, 6 (3 to 12) | 396, 4 (3 to 11) | 765, 5 (2 to 8) |
|  | 59,605 | 50,777 | 110,382 |
| **Pregnancies which resulted in livebirths or resulted in death with neonatal death outcome known** |  |  |  |
| **Neonatal deaths*****; number, rate (95%CI) | 2417, 41 (35 to 48) | 926, 18 (13 to 22) | 3343, 28 (20 to 37) |
| Neonatal deaths by time of death; number, rate (95%CI) |  |  |  |
| Day 0 of life | 1077, 17 (13 to 21) | 402, 7 (5 to 10) | 1479, 12 (7 to 16) |
| Day 1-6 | 765, 14 (11 to 17) | 252, 5 (3 to 6) | 1017, 9 (6 to 11) |
| Day 7-28 | 431, 7 (6 to 9) | 97, 2 (1 to 2) | 528, 4 (2 to 6) |
| Timing unknown | 160, 3 (0 to 8) | 180, 4 (2 to 5) | 340, 3 (2 to 6) |
| * per 100,000 births ** per 1,000 births *** per 1,000 livebirth | | | |

| **Table C in S1 Table: Burden of pregnancy related deaths, stillbirths and neonatal deaths by site** | | | | | | | |
| --- | --- | --- | --- | --- | --- | --- | --- |
|  |  |  |  |  |  |  |  |
|  |  | **Bangladesh** | **DRC** | **Ghana** | **India-UP** | **Kenya** | **Pakistan Karachi** |
| **Pregnancies which lasted 28 weeks or more, or resulted in death with pregnancy related death outcome known** |  | 11316 | 6088 | 12136 | 34979 | 8239 | 3611 |
| Pregnancy-related deaths; number, ratio* (95%CI) |  | 24, 212 (142-315) | 31, 510 (358-723) | 23, 190 (126-285) | 80, 229 (184-285) | 7, 85 (41-178) | 6, 166 (75-369) |
| Pregnancy-related deaths by time of death; number, ratio (95%CI) |  |  |  |  |  |  |  |
| PRD during 3rd trimester |  | 3, 27 (5-78) | 16, 263 (161-429) | - | 18, 51 (32-82) | 2, 24 (6-97) | - |
| PRD during labour/day 0 |  | 17, 150 (88-240) | 3, 49 (16-153) | 2, 16 (4-66) | 32, 91 (65-129) | 1, 12 (2-86) | 5, 138 (58-332) |
| PRD within 2-7 days postpartum |  | 3, 26 (5-77) | - | 1, 8 (1-58) | 10, 29 (15-53) | 1, 12 (2-86) | - |
| PRD within 8-42 days postpartum |  | 1, 9 (0-50) | - | 2, 16 (4-66) | 14, 40 (24-68) | 2, 24 (6-97) | - |
| Timing of PRD unknown |  | 0, 0 (0-0) | 12, 198 (112-347) | 18, 148 (93-235) | 6, 17 (8-38) | 1, 12 (2-86) | 1, 28 (4-196) |
| **Pregnancies which lasted 28 weeks or more, or resulted in death with stillbirth outcome known** |  | 11309 | 6953 | 12810 | 34909 | 8521 | 3629 |
| Stillbirths; number, rate** (95%CI) |  | 469, 41 (38-45) | 149, 21 (18-25) | 354, 27 (25-30) | 1237, 35 (34-37) | 74, 9 (7-11) | 108, 30 (25-36) |
| Stillbirths by time of death; number, rate (95%CI) |  |  |  |  |  |  |  |
| Antepartum stillbirth |  | 101, 9 (8-11) | 50, 8 (6-11) | 46, 13 (10-18) | 646, 19 (17-20) | 25, 3 (2-5) | 65, 18 (14-23) |
| Intrapartum stillbirth |  | 142, 13 (11-15) | 48, 8 (6-11) | 32, 9 (6-13) | 509, 15 (13-16) | 34, 4 (3-6) | 27, 7 (5-11) |
| Timing unknown/verbal autopsy not done |  | 228, 21 (18-23) | 51, 7 (6-10) | 276, 22 (19-24) | 82, 2 (2-3) | 15, 2 (1-3) | 16, 5 (3-7) |
| **Pregnancies which resulted in livebirth, or resulted in death with neonatal death outcome known** |  | 10849 | 6809 | 12478 | 33689 | 8193 | 3523 |
| Neonatal deaths; number, rate*** (95%CI) |  | 403, 37 (34-41) | 144, 21 (18-25) | 256, 21 (18-23) | 1284, 38 (36-40) | 166, 20 (17-24) | 138, 39 (33-46) |
| Neonatal deaths by time of death; number, rate (95%CI) |  |  |  |  |  |  |  |
| NND at day 0 |  | 136, 13 (11-15) | 60, 9 (7-11) | 134, 11 (9-13) | 661, 20 (18-21) | 59, 7 (5-9) | 53, 15 (11-20) |
| NND within 1-6 days of age |  | 122, 11 (9-13) | 35, 5 (4-7) | 78, 6 (5-8) | 388, 12 (10-13) | 23, 3 (2-4) | 54, 16 (12-20) |
| NND within 7-28 days of age |  | 61, 6 (4-7) | 20, 3 (2-5) | 14, 1 (1-2) | 240, 7 (6-8) | 14, 2 (1-3) | 20, 6 (3-9) |
| Timing of NND unknown |  | 90, 8 (7-10) | 29, 4 (3-6) | 30, 2 (2-3) | 1, 0 (0-0) | 72, 9 (7-11) | 13, 3 (2-6) |
| * per 100,000 births ** per 1,000 births. *** per 1,000 livebirths. | | | | | | | |

| **Table C in S1 Table: Burden of pregnancy related deaths, stillbirths and neonatal deaths by site (continued)** | | | | |
| --- | --- | --- | --- | --- |
|  |  |  |  |  |
|  |  | **Pakistan Matiari** | **Tanzania Pemba** | **Zambia** |
| **Pregnancies which lasted 28 weeks or more, or resulted in death with pregnancy related death outcome known** |  | 12139 | 16815 | 6961 |
| Pregnancy-related deaths; number, ratio* (95%CI) |  | 28, 231 (159-334) | 31, 184 (130-262) | 2, 29 (7-115) |
| Pregnancy-related deaths by time of death; number, ratio (95%CI) |  |  |  |  |
| PRD during 3rd trimester |  | 1, 8 (1-58) | 3, 18 (6-55) | - |
| PRD during labour/day 0 |  | 13, 107 (62-184) | 17, 101 (63-163) | 1, 14 (2-102) |
| PRD within 2-7 days post partum |  | 4, 33 (12-88) | 2, 12 (3-48) | - |
| PRD within 8-42 days post partum |  | 7, 58 (27-121) | 9, 54 (28-103) | 1, 14 (2-102) |
| Timing of PRD unknown |  | 3, 25 (8-77) | 0, 0 (0-0) | 0, 0 (0-0) |
| **Pregnancies which lasted 28 weeks or more, or resulted in death with stillbirth outcome known** |  | 12125 | 16811 | 6983 |
| Stillbirths; number, rate** (95%CI) |  | 587, 48 (45-52) | 463, 28 (25-30) | 49, 7 (5-9) |
| Stillbirths by time of death; number, rate (95%CI) |  |  |  |  |
| Antepartum stillbirth |  | 344, 28 (26-31) | 331, 20 (18-22) | 18, 3 (2-5) |
| Intrapartum stillbirth |  | 202, 17 (15-19) | 90, 5 (4-7) | 19, 3 (2-5) |
| Timing unknown/verbal autopsy not done |  | 43, 4 (3-5) | 42, 2 (2-3) | 12, 2 (1-3) |
| **Pregnancies which resulted in livebirth, or resulted in death with neonatal death outcome known** |  | 11544 | 16361 | 6936 |
| Neonatal deaths; number, rate*** (95%CI) |  | 592, 51 (47-55) | 294, 18 (16-20) | 66, 10 (7-12) |
| Neonatal deaths by time of death; number, rate (95%CI) |  |  |  |  |
| NND at day 0 |  | 227, 20 (17-22) | 128, 8 (7-9) | 21, 3 (2-5) |
| NND within 1-6 days of age |  | 201, 17 (15-20) | 91, 6 (4-7) | 25, 4 (2-5) |
| NND within 7-28 days of age |  | 110, 10 (8-11) | 33, 2 (1-3) | 16, 2 (1-4) |
| Timing of NND unknown |  | 56, 5 (4-6) | 44, 3 (2-4) | 5, 1 (0-2) |
| * per 100,000 births ** per 1,000 births. *** per 1,000 livebirths. | | | | |

| **Table D in S1 Table: Adjusted association of background characteristics with pregnancy related death (PRD)** | | | |
| --- | --- | --- | --- |
|  |  |  |  |
|  | **Pregnancy related deaths** | | |
|  | **Number of PRD** | **Risk of PRD per 100,000** (95% CI) | **Adjusted analysis ¹** |
|  |  |  | **Adjusted OR (95%CI)** |
|  |  |  |  |
| **Socio to economic characteristics** | | | |
| **Woman's school attendance** |  |  |  |
| Yes | 118 | 163 (136 to 195) | 1 |
| No | 103 | 284 (234 to 344) | 1.37 (0.94 to 2.00) |
| No data | 11 |  | to |
| **Household wealth quintile** |  |  |  |
| Poorest | 56 | 257 (198 to 334) | 2.31 (1.21 to 4.41) |
| Poor | 48 | 219 (165 to 291) | 2.11 (1.13 to 3.96) |
| Middle | 49 | 225 (170 to 297) | 2.1 (1.13 to 3.92) |
| Rich | 38 | 172 (125 to 237) | 1.8 (0.97 to 3.42) |
| Richest | 25 | 113 (77 to 168) | 1 |
| No data | 16 |  |  |
| **Household access to improved drinking water source*** | | | |
| Yes | 167 | 204 (175 to 237) | 1 |
| No | 53 | 184 (141 to 241) | 0.8 (0.46 to 1.37) |
| No data | 12 |  |  |
| **Household access to toilet facility**** |  |  |  |
| Yes | 126 | 197 (165 to 234) | 1 |
| No | 94 | 202 (165 to 247) | 0.78 (0.41 to 1.64) |
| No data | 12 |  |  |
| **Household use of clean cooking fuel***** | |  |  |
| Yes | 18 | 172 (108 to 272) | 1 |
| No | 202 | 202 (176 to 231) | 0.82 (0.41 to 1.64) |
| No data | 12 |  |  |
| **Women's characteristics and previous obstetric history** |  |  |  |
| **Woman's age (years)** |  |  |  |
| 15 to 19 | 11 | 110 (61 to 198) | 0.56 (0.13 to 2.39) |
| 20 to 24 | 68 | 183 (144 to 232) | 0.93 (0.57 to 1.50) |
| 25 to 29 | 60 | 171 (133 to 220) | 1 |
| 30 to 34 | 46 | 243 (182 to 324) | 1.44 (0.94 to 2.22) |
| 35 to 49 | 35 | 321 (231 to 447) | 1.71 (1.03 to 2.84) |
| No data | 12 |  | to |
| **Number of previous live births)** |  |  |  |
| 0 (first pregnancy) | 55 | 192 (148 to 250) | 1.57 (0.69 to 3.57) |
| 1 | 35 | 154 (110 to 214) | 1 |
| 2 or 3 | 57 | 187 (144 to 242) | 1.12 (0.71 to 1.79) |
| 4+ | 67 | 262 (207 to 333) | 1.23 (0.68 to 3.57) |
| 0 due to pregnancy loss/ stillbirths | 8 | 243 (121 to 484) | 1.57 (0.69 to 3.57) |
| No data | 10 |  |  |
| **Woman's history of miscarriage** |  |  |  |
| Previous pregnancy with no miscarriage | 126 | 191 (160 to 227) | 1 |
| Previous pregnancy with miscarriage (1+) | 39 | 244 (178 to 334) | 1.1 (0.75 to 1.62) |
| No data | 12 |  |  |
| **Woman's history of stillbirth** |  |  |  |
| Previous pregnancy with no stillbirth | 140 | 187 (158 to 220) | 1 |
| Previous pregnancy with stillbirth (1+) | 25 | 349 (236 to 516) | 1.4 (0.89 to 2.23) |
| No data | 12 |  |  |
| **Woman's history of preterm birth** |  |  |  |
| Previous pregnancy with no preterm birth | 157 | 199 (170 to 233) | 1 |
| Previous pregnancy with preterm birth (1+) | 8 | 297 (148 to 592) | 1.27 (0.61 to 2.66) |
| No data | 12 |  |  |
| **Woman's history of C section** |  |  |  |
| Previous pregnancy with no C section | 154 | 195 (167 to 229) | 1 |
| Previous pregnancy with C section (1+) | 13 | 430 (250 to 739) | 2.56 (1.43 to 4.61) |
| No data | 10 |  |  |
| **Multiple birth (index pregnancy)** |  |  |  |
| No | 226 | 202 (177 to 230) | 1 |
| Yes | 6 | 497 (237 to 1039) | 2.75 (1.21 to 6.28) |
| Denominator includes all pregnancies where woman's status at the end of 42days of birth is known. * Includes piped water, public tab, tube well or bottled water. ** Includes flush/pour flush toilet, pit latrine or dry toilet. *** Includes electricity, liquid petroleum gas or kerosene. ¹ Multivariable models include all background characteristics and site. | | | |

| **Table E in S1 Table: Adjusted association of background characteristics with antepartum stillbirths (ASB) and intrapartum stillbirths (ISB)** | | | | | | | |
| --- | --- | --- | --- | --- | --- | --- | --- |
|  | **Antepartum stillbirths** | | |  | **Intrapartum stillbirths** | | |
|  | **Number of ASB** | **Risk of ASB per 1,000 births** (95% CI) | **Adjusted analysis1** |  | **Number of ISB** | **Risk of ISB per 1,000 births** (95% CI) | **Adjusted analysis1** |
|  |  |  | **Adjusted OR (95%CI)** |  |  |  | **Adjusted OR (95%CI)** |
|  |  |  |  |  |  |  |  |
| **Socio to economic characteristics** | | | |  | | | |
| **Woman's school attendance ¹** |  |  |  |  |  |  |  |
| Yes | 837 | 13 (12 to 14) | 1 |  | 587 | 9 (8 to 10) | 1 |
| No | 738 | 22 (21 to 24) | 1.08 (0.93 to 1.25) |  | 477 | 14 (13 to 16) | 1.08 (0.9 to 1.30) |
| No data | 50 |  |  |  | 40 |  |  |
| **Household wealth quintile ¹** |  |  |  |  |  |  |  |
| Poorest | 351 | 18 (16 to 20) | 1.11 (0.90 to 1.37) |  | 235 | 12 (11 to 14) | 1.45 (1.09 to 1.94) |
| Poor | 357 | 18 (16 to 20) | 1.19 (0.97 to 1.45) |  | 232 | 12 (10 to 13) | 1.5 (1.13 to 1.98) |
| Middle | 324 | 16 (15 to 18) | 1.05 (0.86 to 1.28) |  | 252 | 13 (11 to 14) | 1.64 (1.25 to 2.15) |
| Rich | 265 | 13 (12 to 15) | 0.86 (0.70 to 1.06) |  | 212 | 11 (9 to 12) | 1.34 (1.01 to 1.77) |
| Richest | 290 | 14 (13 to 16) | 1 |  | 155 | 8 (7 to 9) | 1 |
| No data | 38 |  |  |  | 18 |  |  |
| **Household access to improved drinking water source* ¹** |  |  |  |  |  |  |  |
| Yes | 1357 | 18 (18 to 19) | 1 |  | 900 | 12 (11 to 13) | 1 |
| No | 234 | 9 (8 to 10) | 0.82 (0.68 to 0.98) |  | 186 | 7 (6 to 8) | 0.77 (0.59 to 1.01) |
| No data | 34 |  |  |  | 18 |  |  |
| **Household access to toilet facility** ¹** |  |  |  |  |  |  |  |
| Yes | 755 | 14 (13 to 15) | 1 |  | 492 | 9 (8 to 10) | 1 |
| No | 836 | 19 (17 to 20) | 0.82 (0.68 to 0.98) |  | 595 | 13 (12 to 14) | 0.92 (0.71 to 1.19) |
| No data | 34 |  |  |  | 17 |  |  |
| **Household use of clean cooking fuel*** ¹** |  |  |  |  |  |  |  |
| Yes | 165 | **17 (15 to 20)** | 1 |  | 84 | 9 (7 to 11) | 1 |
| No | 1426 | 16 (15 to 17) | 1.12 (0.89 to 1.41) |  | 1003 | 11 (10 to 12) | 0.98 (0.71 to 1.35) |
| No data | 34 |  |  |  | 17 |  |  |
| **Woman's characteristics and previous obstetric history** | | | |  | | | |
| **Woman's age (years) ¹** |  |  |  |  |  |  |  |
| 15 to 19 | 85 | 10 (8 to 12) | 0.62 (0.37 to 1.06) |  | 87 | 10 (8 to 12) | 1.19 (0.71 to 1.99) |
| 20 to 24 | 473 | 14 (13 to 15) | 0.90 (0.75 to 1.07) |  | 371 | 11 (10 to 12) | 0.99 (0.80 to 1.23) |
| 25 to 29 | 524 | 16 (15 to 18) | 1 |  | 325 | 10 (9 to 11) | 1 |
| 30 to 34 | 323 | 19 (17 to 21) | 1.19 (1.01 to 1.40) |  | 215 | 13 (11 to 15) | 1.30 (1.07 to 1.59) |
| 35 to 49 | 215 | 23 (20 to 26) | 1.47 (1.21 to 1.78) |  | 100 | 11 (9 to 13) | 1.19 (0.92 to 1.55) |
| No data | 5 |  |  |  | 6 |  |  |
| **Number of previous live births) ¹** |  |  |  |  |  |  |  |
| 0 (first pregnancy) | 417 | 16 (14 to 17) | 1.16 (0.99 to 1.37) |  | 167 | 14 (13 to 15) | 1.8 (1.47 to 2.19) |
| 1 | 299 | 15 (13 to 16) | 1 |  | 367 | 8 (7 to 10) | 1 |
| 2 or 3 | 367 | 13 (12 to 15) | 0.82 (0.70 to 0.97) |  | 232 | 9 (8 to 10) | 1.01 (0.81 to 1.25) |
| 4+ | 415 | 18 (16 to 20) | 0.82 (0.68 to 1.00) |  | 249 | 11 (9 to 12) | 1.35 (1.06 to 1.73) |
| 0 due to pregnancy loss/ stillbirths | 93 | 34 (28 to 41) | 1.38 (1.07 to 1.78) |  | 72 | 26 (21 to 33) | 2.09 (1.53 to 2.85) |
| No data | 34 |  |  |  | 17 |  |  |
| **Woman's history of miscarriage ¹** |  |  |  |  |  |  |  |
| Previous pregnancy with no miscarriage | 813 | 14 (13 to 15) | 1 |  | 531 | 9 (8 to 10) | 1 |
| Previous pregnancy with miscarriage (1+) | 361 | 16 (23 to 28) | 1.41 (1.24 to 1.62) |  | 189 | 13 (12 to 15) | 1.2 (1.00 to 1.44) |
| No data | 34 |  |  |  | 17 |  |  |

| **Table E in S1 Table: Adjusted association of background characteristics with antepartum stillbirths (ASB) and intrapartum stillbirths (ISB) (continued)** | | | | | | | |
| --- | --- | --- | --- | --- | --- | --- | --- |
|  | **Antepartum stillbirths** | | |  | **Intrapartum stillbirths** | | |
|  | **Number of ASB** | **Risk of ASB per 1,000 births** (95% CI) | **Adjusted analysis1** |  | **Number of ISB** | **Risk of ISB per 1,000 births** (95% CI) | **Adjusted analysis1** |
|  |  |  | **Adjusted OR (95%CI)** |  |  |  | **Adjusted OR (95%CI)** |
|  |  |  |  |  |  |  |  |
| **Woman's history of stillbirth ¹** |  |  |  |  |  |  |  |
| Previous pregnancy with no stillbirth | 926 | 14 (13 to 15) | 1 |  | 570 | 8 (8 to 9) | 1 |
| Previous pregnancy with stillbirth (1+) | 248 | 39 (34 to 44) | 2.31 (1.98 to 2.69) |  | 150 | 23 (20 to 27) | 2.16 (1.78 to 2.62) |
| No data | 34 |  |  |  | 17 |  |  |
| **Woman's history of preterm birth ¹** |  |  |  |  |  |  |  |
| Previous pregnancy with no preterm birth | 1092 | 15 (15 to 16) | 1 |  | 690 | 10 (9 to 11) | 1 |
| Previous pregnancy with preterm birth (1+) | 79 | 31 (25 to 39) | 1.57 (1.23 to 2.01) |  | 28 | 11 (8 to 16) | 0.92 (0.62 to 1.38) |
| No data | 37 |  |  |  | 19 |  |  |
| **Woman's history of C section ¹** |  |  |  |  |  |  |  |
| Previous pregnancy with no C section | 1107 | 16 (15 to 17) | 1 |  | 682 | 10 (9 to 10) | 1 |
| Previous pregnancy with C section (1+) | 65 | 25 (19 to 31) | 1.2 (0.92 to 1.56) |  | 36 | 14 (10 to 19) | 1.21 (0.85 to 1.71) |
| No data | 36 |  |  |  | 19 |  |  |
| **Multiple birth (index pregnancy) ¹** |  |  |  |  |  |  |  |
| No | 1573 | 16 (15-16) | 1 |  | 1055 | 10 (10-11) | 1 |
| Yes | 52 | 44 (34-57) | 2.79 (1.99-3.90) |  | 49 | 41 (31-54) | 4.32 (3.02-6.17) |
| Denominator includes all pregnancies where woman's status at the end of 42days of birth is known. * Includes piped water, public tab, tube well or bottled water. ** Includes flush/pour flush toilet, pit latrine or dry toilet. *** Includes electricity, liquid petroleum gas or kerosene. ¹ Multivariable models include all background characteristics and site. | | | | | | | |

| **Table F in S1 Table: Adjusted association of background characteristics with neonatal deaths (NND)** | | | |
| --- | --- | --- | --- |
|  | **Neonatal deaths** | | |
|  |  |  | **Adjusted analysis** |
|  | **Number of NND** | **NND per 1,000 livebirths** (95% CI) | **Adjusted OR** (95%CI) |
| **Socio to economic characteristics** | | | |
| **Woman's school attendance ¹** |  |  |  |
| Yes | 1819 | 25 (24 to 27) | 1 |
| No | 1377 | 39 (38 to 42) | 1.13 (1.01 to 1.26) |
| No data | 114 |  | to |
| **Household wealth quintile ¹** |  |  |  |
| Poorest | 736 | 34 (32 to 37) | 1.38 (1.78 to 1.62) |
| Poor | 670 | 31 (29 to 34) | 1.25 (1.06 to 1.47) |
| Middle | 636 | 29 (28 to 32) | 1.22 (1.04 to 1.43) |
| Rich | 645 | 29 (28 to 32) | 1.27 (1.09 to 1.48) |
| Richest | 526 | 24 (22 to 26) | 1 |
| No data | 97 |  |  |
| **Household access to improved drinking water source* ¹** | | | |
| Yes | 2611 | 32 (32 to 34) | 1 |
| No | 635 | 21 (20 to 24) | 0.79 (0.67 to 0.91) |
| No data | 64 |  |  |
| **Household access to toilet facility** ¹** |  |  |  |
| Yes | 1693 | 26 (26 to 28) | 1 |
| No | 1555 | 34 (33 to 36) | 0.84 (0.93 to 1.34) |
| No data | 62 |  |  |
| **Household use of clean cooking fuel*** ¹** |  |  |  |
| Yes | **305** | 29 (27 to 33) | 1 |
| No | 2942 | 30 (29 to 31) | 1.12 (0.93 to 1.34) |
| No data | 63 |  |  |
| **Woman's characteristics and previous obstetric history** | | | |
| **Woman's age (years) ¹** |  |  |  |
| 15 to 19 | 273 | 27 (24 to 31) | 1.18 (0.87 to 1.60) |
| 20 to 24 | 1114 | 30 (29 to 32) | 1.02 (0.90 to 1.16) |
| 25 to 29 | 990 | 28 (27 to 31) | 1 |
| 30 to 34 | 594 | 32 (30 to 35) | 1.11 (0.99 to 1.25) |
| 35 to 49 | 318 | 30 (27 to 34) | 1.25 (1.07 to 1.45) |
| No data | 21 |  | to |
| **Number of previous live births ¹** | | | |
| 0 (first pregnancy) | 1014 | 36 (34 to 39) | 1.41 (1.28 to 1.56) |
| 1 | 569 | 25 (23 to 28) | 1 |
| 2 or 3 | 830 | 27 (26 to 30) | 1.06 (0.95 to 1.19) |
| 4+ | 700 | 28 (26 to 30) | 1.10 (0.95 to 1.27) |
| 0 due to pregnancy loss/ stillbirths | 135 | 43 (37 to 52) | 1.32 (1.07 to 1.63) |
| No data | 62 |  |  |
| **Woman's history of miscarriage ¹** |  |  |  |
| Previous pregnancy with no miscarriage | 1683 | 25 (25 to 27) | 1 |
| Previous pregnancy with miscarriage (1+) | 551 | 36 (33 to 39) | 1.19 (1.06 to 1.32) |
| No data | 62 |  |  |
| **Woman's history of stillbirth ¹** |  |  |  |
| Previous pregnancy with no stillbirth | 1917 | 26 (25 to 27) | 1 |
| Previous pregnancy with stillbirth (1+) | 317 | 47 (43 to 53) | 1.54 (1.36 to 1.76) |
| No data | 62 |  |  |
| **Woman's history of preterm birth ¹** |  |  |  |
| Previous pregnancy with no preterm birth | 2115 | 27 (26 to 29) | 1 |
| Previous pregnancy with preterm birth (1+) | 106 | 40 (34 to 49) | 1.33 (1.36 to 1.64) |
| No data | 75 |  |  |
| **Woman's history of C section ¹** |  |  |  |
| Previous pregnancy with no C section | 2113 | 27 (26 to 29) | 1 |
| Previous pregnancy with C section (1+) | 116 | 39 (33 to 47) | 1.27 (1.04 to 1.54) |
| No data | 67 |  |  |
| **Multiple birth (index pregnancy) ¹** |  |  |  |
| No | 3115 | 28 (28 to 30) | 1 |
| Yes | 195 | 141 (124 to 161) | 6.4 (5.32 to 7.76) |
| Denominator includes all pregnancies where woman's status at the end of 42days of birth is known. * Includes piped water, public tab, tube well or bottled water. ** Includes flush/pour flush toilet, pit latrine or dry toilet. *** Includes electricity, liquid petroleum gas or kerosene. ¹ Multivariable models include all background characteristics and site. | | | |
