# Supplementary material for: Direct maternal morbidity and the risk of pregnancy-related deaths, stillbirths, and neonatal deaths in South Asia and sub-Saharan Africa: A population-based prospective cohort study in 8 countries
Source: PLoS Med. 2021 Jun 28;18(6):e1003644. doi: 10.1371/journal.pmed.1003644 (PMC8277068; doi:10.1371/journal.pmed.1003644)
Supplement: S1 Text — (DOCX) [file pmed.1003644.s010.docx]

Burden of severe maternal morbidity and association with adverse birth outcomes in sub-Saharan Africa and south Asia: protocol for a prospective cohort study

*AMANHI: Alliance for Maternal and Newborn Health Improvement

**AMANHI Maternal Morbidity Study group**

Bangladesh (Sylhet): Abdullah H Baqui, Dipak Mitra, Mamun Ibne Moin, Nurun Naher, Mohammad Abdul Quaiyum

Democratic Republic of Congo: Antoinette Tshefu, John Otomba, Michel Kalonji, Andre Ngowu, Serge Ngaima

Ghana: Betty Kirkwood, Lisa Hurt, Caitlin Shannon, Samuel Newton, Karen Edmond

India (Shivgarh): Vishwajeet Kumar, Aarti Kumar, Vishwajeet Kumar, Aarti Kumar, Shambhavi Singh, Jai Vir Singh, Amit Kumar Ghosh

Kenya: Fabian Esamai; Irene Marete; Peter Gisore

Pakistan (Karachi): Imran Nisar, Fyezah Jehan, Muhammad Ilyas, Atiya Hussain

Pakistan (Matiari): Sajid Soofi, Shabina Ariff, Yaqub Wasan, Amjid Hussain, Imran Ahmed

Tanzania (Pemba): Sunil Sazawal, Usha Dhingra, Arup Dutta, Said Moh'd Ali, Shaali Makame Ame

Zambia: Davidson Hamer, Katherine Semrau, Fern Hamomba, Bowen Banda, and Julie Herlihy

WHO/MCA: Rajiv Bahl^†^; Alexander Manu^†^; Sachiyo Yoshida

^†^Corresponding authors

# Introduction

Pregnancy and childbirth and their related complications present great risks to the survival, health and well-being of women and their babies globally. Maternal mortality is the most commonly cited maternal health statistic: over 280,000 women die annually from pregnancy-related causes, 85% of these deaths occur in sub-Saharan Africa and South Asia alone.[^1^](#_ENREF_1) The major direct causes of maternal deaths include haemorrhage, infection, unsafe abortion, eclampsia and obstructed labour.[^2^](#_ENREF_2) With each maternal death, however, an additional 20 to 30 women are estimated to suffer acute morbidity and disabilities with substantial impact on their physical, psychological, social and economic functioning.[^3-6^](#_ENREF_3) Approximately 10 million women suffer a spectrum of maternal illnesses ranging in severity from mild disease to acute severe, life-threatening complications or near death events (near misses).[^7-9^](#_ENREF_7) The challenge is that maternal ill-health and their effects are not well defined and seldom measured;[^10^](#_ENREF_10) estimates are imprecise and likely underestimate the true burden,[^3^](#_ENREF_3)^,^ [^10-12^](#_ENREF_10) thereby undermining efforts to harness resources to address them.

Most studies on maternal morbidity are facility-based and in developed countries. One such study among migrant populations in Canada, Australia and other European countries suggested a possible higher baseline risk of severe maternal morbidity (pre-eclampsia, eclampsia and uterine rupture rates) for specific sub-regions. Researchers found that women from sub-Saharan Africa had higher baseline morbidity risk compared to those from other settings.[^13^](#_ENREF_13) However, in low and middle-income countries ( LMICs, especially those in sub-Saharan Africa and South Asia where the burden of morbidity is largest), maternal access to facilities is poor and, therefore, there is a dearth of information on maternal morbidity. Data from the community level, where many births and pregnancy-associated complications occur, are also often lacking. There is a clear need to generate high quality and reliable population-based estimates of severe maternal morbidity using robust epidemiological methods especially in sub-Saharan Africa and Asia.

The Alliance for Maternal and Newborn Health Improvement (AMANHI) maternal morbidity study directly responds to this need. The study aims to describe and quantify severe maternal morbidities and assess their associations with adverse maternal, fetal and newborn outcomes. It is being implemented at nine sites in eight countries of sub-Saharan Africa and South Asia. The study uses harmonized methods to collect prospective population-level maternal morbidity data. AMANHI morbidity study, coordinated by the Maternal, Newborn, Child and Adolescent Health department of the World Health Organization (WHO/MCA) will contribute to improving estimates of severe maternal morbidity; provide a better understanding of the contributory factors that require consideration when designing interventions; and inform the focus of future interventions in order to optimize impact. This manuscript describes the protocol for the harmonized implementation of the study.

# Methodology

## Study design and setting

The AMANHI morbidity study is a population-based, prospective cohort study. Trained AMANHI morbidity study fieldworkers conduct routine surveillance home visits to identify pregnant women, enrol them for follow-up through the pregnancy till after 42 days postpartum to collect data on morbidity, care seeking and outcomes for mothers and babies including preterm birth, intrauterine growth restriction (IUGR), stillbirths and neonatal mortality. It is built on an existing platform of neonatal health studies being implemented in Bangladesh (Sylhet), India (Uttar Pradesh), Pakistan (Karachi and Matiari) in south Asia; and Democratic Republic of Congo (Equator), Ghana (Kintampo), Kenya (Western province), Tanzania (Pemba) and Zambia (Southern Province Zambia) in sub-Saharan Africa. The study spans a period of 24-30 months, with staggered implementation across sites.

**Objectives**

The objectives are to determine the burden of severe acute maternal morbidity, describe the care received by pregnant and delivered women, and examine the association of severe maternal morbidity and care received with adverse maternal, fetal and neonatal outcomes.

## Study Population and Setting

The AMANHI morbidity study is being implemented in predominantly rural populations where women’s educational levels are low. Families mainly engage in subsistence agriculture, petty trading and fishing. A variety of health facilities ranging from community clinics (providing only first aid and referral services) to district hospitals serve the population. In AMANHI, these health facilities were mapped according to their type (health post/community clinic, health centre, district or provincial hospital) and range of services provided (out-patients only; basic delivery services; basic or comprehensive emergency obstetric care). This mapping was done as part of on-going community-based pregnancy and birth surveillance that involves 1-3 monthly household visits by trained fieldworkers to all women of reproductive age (15 to 49 years). The exception to the community surveillance is Zambia where recruitment is facility-based as explained below. With each woman visited at least once every three months, pregnancies are identified early and any complications or adverse outcomes are documented close to when they occur. Any woman of reproductive age who resides in the study area is eligible for enrolment into the study once they fall pregnant and consent to participate. In order to generate comparable data that will be amenable to pooled analyses, the implementation of the study is harmonised across sites as described in the following sections.

**Harmonization of protocols and implementation strategies**

When the study was planned in 2012, investigators from all participating sites agreed on common protocols, standard operating procedures, methods and strategies for implementation.

***Protocols***

The principal investigators put together an agreed common protocol for the study. They developed an initial generic protocol from which all the sites developed specific adaptations for their sites. These protocols were submitted to ethical review committees of the WHO and at the respective sites.

***Standard operating procedures & implementing strategies***

1. *Core variable tables*: The AMANHI investigators discussed common data to collect and collated these into a core variable table (CVT) to be used across sites. The table specifies and defines signs and symptoms elicited during interviews with women, important exposure variables such as maternal age, education, etc., and how data is stored.
2. *Timing and frequency of visits*: The timing and number of visits are also harmonized, as shown in figure 1, and so chosen to enable detailed information on women’s morbidity experiences, within each trimester of pregnancy, to be collected close to their occurrence. After enrolment, trained fieldworkers (with a minimum of 10 years formal education) follow all pregnant women at baseline, 24-28 weeks, 32-36 weeks and after 37 weeks of gestation for morbidity data collection. Sites estimate gestational ages of pregnancies using women’s reported date of last normal menstrual period (LMP) to plan the antenatal visits. At the end of the pregnancy, fieldworkers make postnatal visits within a week and post 42 days to collect pregnancy outcomes, morbidities and their outcomes as well as care sought for the mother and baby.
3. *Training of data collectors and quality assurance*: The WHO/MCA trained and standardized AMANHI investigators from all the sites on the strategy for uniform implementation of the study across sites. These in turn trained study fieldworkers at their respective sites. The team also agreed on a common process for monitoring implementation and data quality across sites by the WHO/MCA.

***Study supplies/equipment***

Fieldworkers are provided equipment and training to directly assess pregnant women for hypertensive disorders during home visits. Each fieldworker uses urinalysis kits (Uristix^®^ by Siemens, Gujarat, India) to assess proteinuria and a digital sphygmomanometer (Microlife^®^ WatchBP^®^ Home A BP3MX1-3, Widnau, Switzerland)[^14^](#_ENREF_14)^,^ [^15^](#_ENREF_15) to measure women’s blood pressure. All these study materials were procured from a common source.

## Surveillance for pregnancy identification

During home visits, fieldworkers use a variety of methods to identify pregnant women. These include direct disclosure by women or eliciting information on missed menstrual periods from women’s LMPs. When unsure, women in Bangladesh, Pakistan (Karachi and Matiari), India (UP) and Tanzania (Pemba) had the option to request a urine pregnancy test to confirm pregnancies. Zambia is the exception where, because over 96% of women in the study area attend antenatal care (ANC) clinics during pregnancy,[^16^](#_ENREF_16) recruitment into AMANHI is done at these ANC clinics. A common information sheet containing comprehensive summary of the study objectives, risks and benefits is read to potential participants in their local or their preferred language to help them make informed decision to participate in the study. Consented mothers receive a unique study identification number (study ID).

## Follow-up on enrolled women

***Home visits***

The AMANHI morbidity study employs both active and passive surveillance for collecting maternal morbidity data. In each site, fieldworkers actively collect data on women’s self-reported morbidity and directly assess for hypertensive disorders during pregnancy and postpartum visits. These data are augmented with abstraction of linked data from health facilities where women go to deliver or seek care for pregnancy or postpartum morbidity. For all births, fieldworkers also interview birth attendants to obtain additional details on complications during labour and delivery. The unique study ID provided to each enrolled woman is used to link data from these various sources. At baseline/enrolment fieldworkers collect household characteristics and baseline socio-demographic data on participants. They conduct an assets inventory for classifying households into socio-economic quintiles. This will be used to evaluate inequities in the distribution of severe maternal morbidity in the AMANHI cohort. They also collect previous medical and obstetric, history of cigarette smoking or alcohol ingestion and collect data on morbidity experiences since the onset of the pregnancy. They pre-inform women that AMANHI will collect their data at health facilities anytime they attend during the pregnancy or after birth. They record women’s study IDs on health facility cards for easy reference.

During the first antenatal home visit (24-28 weeks), fieldworkers first ascertain the status of the pregnancy and collect data on morbidities experienced. If the woman has commenced routine ANC clinic attendance within the regular health system, fieldworkers abstract data on morbidity, results of laboratory investigations (e.g. haemoglobin level and presence of malaria parasites), ultrasound examinations and maternal anthropometric measures (height, weight, mid upper arm circumference) taken by health professionals from ANC cards. At the end they measure women’s blood pressure and check their urine for proteins. At subsequent ANC visits, the same form is used to collect morbidity data on morbidity experiences in the interval between the previous and the index visit.

1. *Reported morbidity*: Study fieldworkers ask questions around morbidities during the pregnancy. These questions are to elicit any occurrence of severe maternal morbidity notably haemorrhage (antepartum and postpartum), infections, abortion complications, prolonged/obstructed labour, fistulae, severe anaemia, signs of pre-eclampsia or eclampsia, etc. For each morbidity, an assessment is made of the timing of onset, the severity and any interventions received and from where.
2. *Measurements*: In addition, at every home visit, trained fieldworkers directly measure women’s blood pressure (BP) and test their urine for proteins as part of active assessment for hypertensive disorders of pregnancy. At all visits, women with high blood pressure (systolic blood pressure>140mmHg or diastolic pressure>90mmHg) are referred to participating health facilities for appropriate care.

If the pregnancy has been aborted/miscarried, they terminate the AMANHI pregnancy follow-up and complete postnatal forms for the woman. At the first postnatal visit, data are collected on women’s reported morbidity during labour, delivery and immediately after birth including care seeking and outcomes for mother and baby. Fieldworkers also abstract morbidity data and the birthweight of babies from available health facility records (hospital folders, postnatal clinic record cards, etc.) during the postnatal visits.

During home visits, data on premature births, intra-uterine growth retardation (IUGR) and mortality outcomes are also collected from this cohort. Fieldworkers conduct verbal autopsies (VAs) for all deaths of woman of reproductive age, fetus or neonate using standardized tools and procedures. Harmonised protocols are used by trained physicians to confirm timing, type and to assign causes of these deaths based on principles of the International Classification of Diseases.

To corroborate women’s reported morbidity experiences during home visits, the following additional surveillance activities are conducted in tandem with the home visits:

## Outcomes

The main outcome of the study is the prevalence of severe acute maternal morbidity (operationally defined to include acute problems suffered during pregnancy, through childbirth to the end of 42 days postpartum. Severe acute maternal morbidity will include, but is not limited to, pre-(eclampsia), antepartum and postpartum haemorrhage, abortion complications, maternal infections, obstructed labour and other complications arising out of these. Denominators for rate estimates will be total pregnancies or the number of women who become pregnant among the cohort whilst those who suffer any severe acute morbidity will contribute data to the numerators. With the AMANHI surveillance system, these denominators and numerators will guarantee estimation of reliable morbidity burden estimates. In estimating prevalence of hypertensive disorders for which AMANHI is directly assessing women’s blood pressure and urine proteins at baseline (pre-pregnancy levels) and after 42 days postpartum (when those who developed pregnancy-induced hypertension will have returned to baseline states), it will be possible to describe a wide spectrum of hypertensive disorders including the classical pregnancy induced hypertension where women are normotensive pre-pregnancy, develop pregnancy-induced hypertension and return to normotensive state after delivery. Care seeking and care given for each morbidity will be described.

## Sample size considerations

The sample size contributions from each of the sites are as shown **in table 2**. The 160,000 total participants in the study are sufficient for assessing association of severe maternal morbidity with adverse maternal, fetal and neonatal outcomes based on an assumption that all individual sites should have adequate power to detect association between preterm birth and any morbidity with a prevalence of 7.5% or more. Data will be pooled across sites for evaluating morbidities with lower prevalence, especially in assessing associations with stillbirths and early neonatal deaths.

## Data management

***Data processing:*** The study uses paper forms or tablet-based software for data collection. Forms are independently double entered by two clerks into study databases with stringent range and consistency (R&C) checks with the exception of Zambia where field monitors collected data using forms designed in the TeleForms^®^ system (HP, Cambridge, UK). After Zambian supervisors reviewed the forms for completeness, they were scanned, entered, and exported into a Microsoft^®^ Access database. Similar R&C checks are built into the software used for data capture at sites using tablets. Data managers within the sites conduct inter-database checks to reconcile and synchronize data from various forms using the woman’s unique study ID as the link. Cleaned data are saved on special study servers with password-protected access to only principal investigators in the sites. They generate data back-ups on external drives at regular intervals. Every three months, sites transfer back-up data to a dedicated server at the WHO/MCA for external quality control and storage.

***Data analyses:*** Analyses will be done using Stata^®^ statistical software package.[^17^](#_ENREF_17) Incidence of severe maternal morbidities will be estimated. The burden of adverse birth outcomes will also be estimated. Associations will be independently explored between various maternal characteristics (confounders) such as socio-economic status, educational attainment, age, parity, etc. and severe maternal morbidity as well as the adverse birth outcomes. The effect of exposure to severe acute maternal morbidity on adverse birth outcomes will be estimated using appropriate regression models. Test of interaction will be done to assess effect modification of treatment received by study women on association between severe maternal morbidities and adverse pregnancy, birth and neonatal outcomes.

## Quality Monitoring

The WHO/MCA centrally coordinates and monitors the harmonized implementation and quality of fieldwork and data in the AMANHI morbidity study. Individual sites send monthly fieldwork progress reports to WHO/MCA, highlighting their key challenges. At quarterly intervals, the WHO/MCA team run quality control checks on all transferred data to identify outliers and provide feedback to the sites. Data are also reconciled with the monthly fieldwork progress reports to check consistency. WHO/MCA sends experts to the sites once or twice each year to assess progress and quality of implementation, provide technical input and to enhance the harmonized implementation. They also discuss challenges with the sites and provide a detailed report to the WHO/MCA highlighting key issues of benefit to and for follow-up with the other sites.

## Ethical considerations

All women are individually consented to participate in the AMANHI morbidity study. Local and institutional ethics committees from all nine sites approved the AMANHI study protocols. The Ethics Review Committee of the WHO has also approved a combined master protocol with components on the role of the WHO/MCA.

## Dissemination Plan

The results of the study will be disseminated among the public health, maternal and newborn health community of researchers, policy-makers and programme managers. Channels for dissemination will include peer-reviewed journals, print and electronic media and academic presentations (oral and poster) at appropriate fora. In each participating country, there will be extensive briefing on their country-specific and overall study results, and the team of researchers and stakeholders will discuss implications of the study for interventions and programmes in those settings.

# Discussion

Inadequate attention to reducing the burden of maternal morbidity may be contributing to the slow progress in reducing preventable maternal mortality.[^18^](#_ENREF_18) Beyond survival, another significant statistic is the number of women who develop severe acute morbidities and/or severe chronic disabilities that are incompatible with normal physical, psychological or economic viability and who are abandoned by loved ones, families, friends and society.[^4-6^](#_ENREF_4) The biggest hurdle to planning and delivery of effective interventions is the dearth of data on maternal morbidities. Good quality data are essential for strategic planning and targeting of interventions. In LMICs of sub-Saharan Africa and South Asia where resources are limited and a disproportionate burden of severe acute maternal morbidities exists, evidence-based data-driven strategic prioritization of investments and resource allocation to address these is paramount.[^3^](#_ENREF_3)^,^ [^4^](#_ENREF_4)^,^ [^19^](#_ENREF_19)^,^ [^20^](#_ENREF_20)

The AMANHI maternal morbidity study will generate reliable estimates of severe maternal morbidity from one of the largest population-based, multi-country studies in sub-Saharan Africa and South Asia. AMANHI has many advantages; implementation is being harmonized across sites and common definitions of severe maternal morbidity are being used. This will ensure comparability of data and facilitate pooled analyses across sites. The sample size is large and with the active pregnancy and birth surveillance allowing for accurate denominators, estimates generated will be precise and reliable. The combined comparative advantages of large sample size and homogeneity in the data across sites will additionally allow for analyses on very rare outcomes.

AMANHI will provide the dual benefit of a unique opportunity to assess associations between various exposures, severe maternal morbidity and adverse pregnancy outcomes and also address the gap in the availability of quality data for validation of model-based estimates. The data will also form the baseline for generation of more accurate estimates of the real impact of severe acute morbidities on health and well-being of women after pregnancy and childbirth.

While this contribution of reliable and good quality data on maternal morbidity from the AMANHI study to global public health is significant in that it will inform policy direction, interventions and programmes, we do recognise that it remains the first step needed to create a sustainable platform for prioritization and ensuring equitable coverage of maternal health interventions for the benefit of both mothers and their newborns.

## References

1. World Health Organization. Trends in maternal mortality 1990‐2013 <http://apps.who.int/iris/bitstream/10665/112682/2/9789241507226_eng.pdf?ua=1>. 2014.

2. Ribeiro PS, Jacobsen KH, Mathers CD, Garcia-Moreno C. Priorities for women's health from the Global Burden of Disease study. *International journal of gynaecology and obstetrics: the official organ of the International Federation of Gynaecology and Obstetrics* 2008; **102**(1): 82-90.

3. Prual A, Bouvier-Colle MH, de Bernis L, Breart G. Severe maternal morbidity from direct obstetric causes in West Africa: incidence and case fatality rates. *Bulletin of the World Health Organization* 2000; **78**(5): 593-602.

4. Murray C, Lopez A, editors. Health Dimensions of Sex and Reproduction. Boston: Boston: Harvard University Press,; 1998.

5. Pacagnella RC, Cecatti JG, Camargo RP, et al. Rationale for a long-term evaluation of the consequences of potentially life-threatening maternal conditions and maternal "near-miss" incidents using a multidimensional approach. *Journal of obstetrics and gynaecology Canada : JOGC = Journal d'obstetrique et gynecologie du Canada : JOGC* 2010; **32**(8): 730-8.

6. Reichenheim ME, Zylbersztajn F, Moraes CL, Lobato G. Severe acute obstetric morbidity (near-miss): a review of the relative use of its diagnostic indicators. *Archives of gynecology and obstetrics* 2009; **280**(3): 337-43.

7. World Health Organization. Why do so many women still die in pregnancy or childbirth? <http://www.who.int/features/qa/12/en/>. 2014 (accessed March 26 2015).

8. Say L, Souza JP, Pattinson RC, Mortality WHOwgoM, Morbidity c. Maternal near miss--towards a standard tool for monitoring quality of maternal health care. *Best practice & research Clinical obstetrics & gynaecology* 2009; **23**(3): 287-96.

9. Mantel GD, Buchmann E, Rees H, Pattinson RC. Severe acute maternal morbidity: a pilot study of a definition for a near-miss. *British journal of obstetrics and gynaecology* 1998; **105**(9): 985-90.

10. Koblinsky M, Chowdhury ME, Moran A, Ronsmans C. Maternal morbidity and disability and their consequences: neglected agenda in maternal health. *Journal of health, population, and nutrition* 2012; **30**(2): 124-30.

11. Kuklina EV, Whiteman MK, Hillis SD, et al. An enhanced method for identifying obstetric deliveries: implications for estimating maternal morbidity. *Maternal and child health journal* 2008; **12**(4): 469-77.

12. Say L, Chou D, Gemmill A, et al. Global causes of maternal death: a WHO systematic analysis. *The Lancet Global health* 2014; **2**(6): e323-33.

13. Urquia ML, Glazier RH, Mortensen L, et al. Severe maternal morbidity associated with maternal birthplace in three high-immigration settings. *Eur J Public Health* 2015.

14. Stergiou GS, Giovas PP, Gkinos CP, Patouras JD. Validation of the Microlife WatchBP Home device for self home blood pressure measurement according to the International Protocol. *Blood pressure monitoring* 2007; **12**(3): 185-8.

15. Chung Y, de Greeff A, Shennan A. Validation and compliance of a home monitoring device in pregnancy: microlife WatchBP home. *Hypertens Pregnancy* 2009; **28**(3): 348-59.

16. Central Statistical Office (CSO) [Zambia], Ministry of Health [Zambia], ICF International. Zambia Demographic and Health Survey 2013-14 Rockville, Maryland, USA: Central Statistical Office [Zambia], 2014.

17. StataCorp. Stata Statistical Software: Release 11.2. College Station, TX: StataCorp; 2009.

18. Tinker A, Koblinsky M. Making motherhood safe. Washington DC: World Bank, 1993.

19. Vandecruys HI, Pattinson RC, Macdonald AP, Mantel GD. Severe acute maternal morbidity and mortality in the Pretoria Academic Complex: changing patterns over 4 years. *European journal of obstetrics, gynecology, and reproductive biology* 2002; **102**(1): 6-10.

20. Sahel A, Brouwere VD, Lardi M, Lerberghe WV, Ronsmans C, Filippi V. [Obstetric catastrophes barely just avoided: near misses in Moroccan hospitals]. *Sante* 2001; **11**(4): 229-35.

Day of birth

| Enrolment  (< 24 weeks) | AN visit 1 (24-28 weeks) | AN visit 2 (32-36 weeks) | AN visit 3 (38+ weeks) | PN visit 1 (1-6 days after birth) | | PN visit 2 (42-60 days after birth) | |  |
| --- | --- | --- | --- | --- | --- | --- | --- | --- |
|  | | | | |  |  |  |  |
|  | | | | | | |  |  |
| Pregnancy outcome/birth surveillance | | | | Postpartum morbidity surveillance | | | | |

Figure 1. Antenatal (AN) and postnatal (PN) follow-up visit schedule - AMANHI morbidity study

**Table 1. Summary of data collected at various visits in the AMANHI maternal morbidity study**

| **Main category** | **Thematic areas of data collection** | | **Source of data** | **Visit & Time of data collection** |
| --- | --- | --- | --- | --- |
| Maternal morbidity | 1. Antepartum haemorrhage 2. Postpartum haemorrhage 3. Hypertensive disorders of pregnancy 4. Difficulty in labour 5. Infection 6. Obstetric fistula | 1. Maternal self-report 2. Maternal self–report 3. Measurements of blood pressure and urine protein at all home visits 4. Maternal self-reporting 5. Maternal self-reporting 6. Maternal self-reporting | | Antenatal home visits (24–28 weeks, 32–36 weeks, 37–40 weeks), postnatal home visits (1–6 days after birth and 42– 60 days after birth) |
| Background characteristics | Demographic, socio-economic, other characteristics of the woman and her household and an asset inventory to be used in constructing an asset index for classifying women into wealth quintiles | Maternal self-reports | | Baseline home visit at enrolment |
| Medical history | Previous obstetric and gynaecological history, history of birth defects and congenital anomalies among previous babies, stillbirths and IUGRs; previous medical and surgical history including medicinal prescription drugs taken or being taken for chronic diseases. | | Maternal self-reports and health facility records | Baseline home visit at enrolment |
| Risk factors and exposures | Cigarette smoking, alcohol ingestion, smoke from biomass cooking fuels | | Maternal self-reports | Baseline home visit at enrolment |
| Anthropometry | Paternal and maternal weights and heights, maternal mid-upper arm circumference. | | Health facility records | All antenatal and postnatal home visits |

**Table 2. Expected number of participants to be enrolled from the AMANHI sites (by region) and precision that can be obtained around estimates**

| **Region** | **Study country** | **Sample Size** | **Expected width of 95% CI if prevalence of morbidity = 2%** | **Relative Precision** |
| --- | --- | --- | --- | --- |
| **Sub-Saharan Africa** | DRC | 20000 | 1.8% to 2.2% | +10% |
|  | Ghana | 10000 | 1.7% to 2.3% | +14% |
|  | Kenya | 20000 | 1.8% to 2.2% | +10% |
|  | Tanzania (2 sites) | 15000 | 1.8% to 2.2% | +11% |
|  | Zambia | 25000 | 1.8% to 2.2% | +9% |
|  | **Pooled** | **90,000** | **1.9% to 2.1%** | **+5%** |
| **South Asia** | Bangladesh | 19000 | 1.8% to 2.2% | +10% |
|  | India | 35000 | 1.9% to 2.1% | +7% |
|  | Pakistan (2 sites) | 16000 | 1.8% to 2.2% | +11% |
|  | **Pooled** | **70,000** | **1.9% to 2.1%** | **+5%** |

**STUDY INSTRUMENT**

**OBJECTIVE 2 CORE VARIABLES BY FORM**

| **BASELINE FORM (completed at the first antenatal visit)** | | | | | | |
| --- | --- | --- | --- | --- | --- | --- |
| **No.** | | **Question** | **Outcomes** | **Variable Name** | **Field Type** | **Size** |
|  | | Date of filling the form  (all dates dd/mm/yyyy) | 01/06/2012 to 30/04/2013 | DATEVISIT | Date | 8 |
|  | | Study site | 11=Bangladesh, 12=DRC, 13=Ghana, 15=India Shivgarh, 16=Kenya, 17=Pakistan Karachi, 18=Pakistan Matiari, 20=Tanzania Pemba, 21=Zambia | SITE | Numeric | 2 |
|  | | Worker code | Each site to define valid range | FW | Text | 3 |
|  | | Woman ID | Each site to define valid range | WHOID | Text | 10 |
|  | | Has the woman given her consent to be enrolled in the Cohort study? | 1=yes, 2=no | CONSENT | Numeric | 2 |
| **PREGNANCY HISTORY** | | | | | | |
|  | | Including this pregnancy, how many times in your life have you been pregnant? | xx-yy, 88=don’t know | GRAVIDITY | Numeric | 2 |
|  | | How many deliveries of a live child have you had? | xx-yy, 88=don’t know; 99=NA, first pregnancy | PARITY | Numeric | 2 |
|  | | How many deliveries of a stillborn child have you had (e.g., the baby did not cry or move or breathe after delivery) | xx-yy, 88=don’t know; 99=NA, first pregnancy | PREVSB | Numeric | 2 |
|  | | How many pregnancies have you had which ended before 6 months? | xx-yy, 88=don’t know; 99=NA, first pregnancy | PREVMIS | Numeric | 2 |
|  | | How many living children do you have? | xx-yy, 88=don’t know; 99=NA, first pregnancy | ALIVECHILD | Numeric | 2 |
|  | | How many of your children have died? | xx-yy, 88=don’t know; 99=NA, first pregnancy or no live born children | DIEDCHILD | Numeric | 2 |
|  | | Did you attend ANC for all, some or none of your previous pregnancies? | 11=all; 12=some; 13=none; 99=NA, first pregnancy | PREVANCSK | Numeric | 2 |
|  | | In any of your previous pregnancies, did you have severe bleeding or haemorrhaging before delivery? | 1=yes, 2=no; 9=NA, first pregnancy | PREVHEMOAP | Numeric | 1 |
|  | | In any of your previous pregnancies, did you have severe bleeding or haemorrhaging after delivery? | 1=yes, 2=no; 9=NA, first pregnancy | PREVHEMOPP | Numeric | 1 |
|  | | In any of your previous pregnancies, did you have fits or convulsions? | 1=yes, 2=no; 9=NA, first pregnancy | PREVCONV | Numeric | 1 |
|  | | In any of your previous pregnancies, did your water break more than 24 hours (one day) before labour pains started? | 1=yes, 2=no; 9=NA, first pregnancy | PREVPROM | Numeric | 1 |
|  | | In any of your previous pregnancies, were you admitted to stay overnight in the hospital three or more nights? | 1=yes, 2=no; 9=NA, first pregnancy | PREVPADM | Numeric | 1 |
|  | | In any of your previous pregnancies, did you deliver twins or triplets? | 1=yes, 2=no; 9=NA, first pregnancy | PREVMULTIP | Numeric | 1 |
|  | | In any of your previous pregnancies, did you have a caesarean section? | 1=yes, 2=no; 9=NA, first pregnancy | PREVCS | Numeric | 1 |
|  | | Did any of your previous pregnancies end more than 1 month before time? | 1=yes, 2=no; 9=NA, first pregnancy | PREVPREM | Numeric | 1 |
|  | | In any of your previous pregnancies, have any other serious problem that I haven’t asked you about? | 1=yes, 2=no; 9=NA, first pregnancy | PREVOTHER | Numeric | 1 |
|  | | If yes, other serious problems, specify: | Text | SPREVOTHER | Text | 50 |
|  | | In the year before THIS pregnancy, did you have any health problems? | 1=yes, 2=no | PROBBFPREG | Numeric | 1 |
|  | | If yes, health problems in year before pregnancy, specify: | Text | SPROBBF | Text | 50 |
|  | | Has a doctor every told you that you have a medical problem involving your private parts/pelvis or bladder while not pregnant? | 1=yes, 2=no | PREVFIST | Numeric | 1 |
|  | | Have you ever undergone surgery in the private parts, pelvis or bladder, not related to pregnancy (i.e. surgery other than caesarean section, abortion or surgery for contraception)? | 1=yes, 2=no | OREVOPFIS | Numeric | 1 |
|  | | Have you ever had a fit / convulsion when you were not pregnant? | 1=yes, 2=no | NPREGCONV | Numeric | 1 |
|  | | Has a doctor ever told you that you have epilepsy? | 1=yes, 2=no | EPILEPSY | Numeric | 1 |
| **BACKGROUND DETAILS MOTHER** | | | | | | |
|  | | What is your age? | xx-yy, 88=don’t know | MOTHERAGE | Numeric | 2 |
|  | | Place woman in an age category: | 11=15-19 years; 12=20-24 years; 13=25-29 years; 14=30=34 years; 15=35-39 years; 16=40-44 years; 17=45-49 years. | ATTENDANT1 | Numeric | 2 |
|  | | How many years of schooling did you complete? | 00 to 25, 99=not applicable | MOTHEREDU | Numeric | 2 |
|  | | What is your current occupation? | 11= government job, 12= private job, 13= self employed, 14= farming only, 15= daily wage earner, 16= other work, 17= does not work, , 99=not applicable | MOTHEROCC | Numeric | 2 |
|  | | If occupation is "other work", specify | Text | MOCCOTH | Text | 50 |
| **BACKGROUND DETAILS HOUSEHOLD** | | | | | | |
|  | | Who is the head of your household? | 11= Mother of baby, 12= Father of baby, 13= Grandmother of baby, 14= Grandfather of baby, 15= other | HEAD | Numeric | 2 |
|  | | How many years of schooling did the baby’s father of the baby or head of the household complete? | 00 to 25, 99= not applicable 88= not known | FATHEREDU | Numeric | 2 |
|  | | What is the current occupation of the father or the head of the household? | 11= government service, 12= private service, 13= daily wage earner, 14= self employed, 15= farming, 16= does not work, 17= other, 99=not applicable | FATHEROCC | Numeric | 2 |
|  | | If occupation of baby’s father or head of the household is "other", specify | Text | FOCCOTH | Text | 20 |
|  | | Is baby’s father currently staying with the family? | 1=yes, 2= no, 9=not applicable | FATHSTAY | Numeric | 1 |
|  | | What is the main source of drinking water for members of your household? | 11= piped water into the dwelling, 12= public tap, 13= Tube well or borehole or hand pump, 14= Open well, 15= closed well, 16= Tanker truck, 17= small cart with tank, 18= Surface water (river / dam / lake / pond / stream / canal), 19= bottled water, 20= rain water, 21= other | HOUSEWTR | Numeric | 2 |
|  | | If source of water is "other", specify | Text | WTROTHER | Text | 20 |
|  | | What kind of toilet facility do you usually use? | 11= Flush or pour flush toilet, 12= Pit latrine, 13= dry toilet, 14= bucket latrine, 15= No toilet facility (uses open space or field), 16= other | TOILET | Numeric | 2 |
|  | | If toilet facility is "other", specify | Text | TOILETOTH | Text | 20 |
|  | | What is the religion of the head of the household? | 11= Christian, 12= Muslim, 13= Hindu, 14= none, 15= Traditional African, 16=other | HHRELIG | Numeric | 2 |
|  | | If religion "other", specify | Text | RELIGOTH | Text | 20 |
|  | | What is the ethnic group of the head of the household? | **Each site to decide possible outcomes** | **XX_ETHNIC** | Numeric | 2 |
|  | | What type of fuel does the household mainly use for cooking | 11= electricity, 12= liquid petroleum gas / natural gas, 13= kerosene, 14= coal / lignite, 15= charcoal, 16= wood, 17= straw /shrub / grass, 18= agricultural crop waste, 19= dung cakes, 20= biogas, 21= other | FUELCOOK | Numeric | 2 |
|  | | If cooking fuel is "other", specify | Text | FUELOTH | Text | 20 |
|  | | How many rooms in your house (including the kitchen) are used for sleeping? | 01 to 20 | ROOMS | Numeric | 2 |
|  | | How many persons slept in the house last night? | 01 to 40 | PERSONS | Numeric | 2 |
| **ASSETS (to be used for PCA in determining wealth quintiles)**  **QUESTIONS TO BE DECIDED BY EACH SITE. YOU CAN USE THE COUNTRY DHS SURVEY QUESTIONNAIRE**  **SEE EXAMPLE QUESTIONS BELOW FOR GHANA** | | | | | | |
| 50 | Do you own or rent the house you live in, or do you have another type of arrangement, such as “perching”? | | 11= Sole Ownership, 12= Joint Ownership, 13= Renting, 14= Family/relative’s house, 15= House provided rent free, 16= Perching, 17= Other, 88= NK | GH_OWNHOUSE | Numeric | 2 |
| 51 | Does this household own any land? | | 1= Yes, 2= no | GH_OWNLAND | Numeric | 1 |
|  | **Does anyone in the household own:** | |  |  |  |  |
| 52 | Is there electrical supply at home? | | 1. Yes, 2. No | GH_ELECTR | Numeric | 1 |
| 53 | Chickens | | 1. Yes, 2. No | GH_CHICKEN | Numeric | 1 |
| 54 | Sheep | | 1. Yes, 2. No | GH_SHEEP | Numeric | 1 |
| 55 | Other animals | | 1. Yes, 2. No | GH_OTHANIMAL | Numeric | 1 |
| 56 | Mattress | | 1. Yes, 2. No | GH_MATTRESS | Numeric | 1 |
| 57 | Stove or cooker | | 1. Yes, 2. No | GH_PRESCOOK | Numeric | 1 |
| 58 | Chair | | 1. Yes, 2. No | GH_CHAIR | Numeric | 1 |
| 59 | Cot or bed | | 1. Yes, 2. No | GH_BED | Numeric | 1 |
| 60 | Divider | | 1. Yes, 2. No | GH_DIVIDER | Numeric | 1 |
| 61 | Table | | 1. Yes, 2. No | GH_TABLE | Numeric | 1 |
| 62 | Electric fan | | 1. Yes, 2. No | GH_FAN | Numeric | 1 |
| 63 | Radio | | 1. Yes, 2. No | GH_RADIO | Numeric | 1 |
| 64 | Television | | 1. Yes, 2. No | GH_BWTV | Numeric | 2 |
| 65 | Sewing machine | | 1. Yes, 2. No | GH_SEW | Numeric | 1 |
| 66 | Mobile telephone | | 1. Yes, 2. No | GH_MOBILE | Numeric | 1 |
| 67 | mosquito net | | 1. Yes, 2. No | GH_MOSNET | Numeric | 1 |
| 68 | Computer | | 1. Yes, 2. No | GH_COMP | Numeric | 1 |
| 69 | refrigerator | | 1. Yes, 2. No | GH_FRIDGE | Numeric | 1 |
| 70 | Watch or clock | | 1. Yes, 2. No | GH_WATCH | Numeric | 1 |
| 71 | Bicycle | | 1. Yes, 2. No | GH_BICYCLE | Numeric | 1 |
| 72 | Motor cycle or scooter | | 1. Yes, 2. No | GH_MOTOCY | Numeric | 1 |
| 73 | Animal-drawn cart | | 1. Yes, 2. No | GH_ANIMCART | Numeric | 1 |
| 74 | Car | | 1. Yes, 2. No | GH_CAR | Numeric | 1 |
| 75 | Thresher | | 1. Yes, 2. No | GH_THRSHR | Numeric | 1 |
| 76 | Tractor | | 1. Yes, 2. No | GH_TRACTOR | Numeric | 1 |
| 78 | **Materials used in the construction of the house (observe):** | |  |  |  |  |
| 79 | Flooring in sleeping rooms | | 11=Cement, 12= Mud/clay | GH_FLOOR | Numeric | 2 |
| 80 | Roof of household | | 11=Metal, 12=Asbestos, 13=Thatch; 14=Mud; 15=Other | GH_ROOF | Numeric | 2 |
| 81 | Wall of household | | 11=Cement, 12=Mud, 13=Other | GH_WALL | Numeric | 2 |

| **ANTENATAL FORM (24-28 WEEKS, 32-36 WEEKS, 38-40 weeks)** | | | | | |
| --- | --- | --- | --- | --- | --- |
| **No.** | **Question** | **Outcomes** | **Variable Name** | **Field Type** | **Size** |
| 1 | Date of filling the form  (all dates dd/mm/yyyy) | 01/06/2010 to 30/04/2013  (sites to specify date range) | ADATEVISIT | Date | 8 |
| 2 | Number of visit | 1=24-28 weeks gestation, 2=32-36 weeks gestation, 3= 38-40 weeks gestation | NUMANVISIT | Numeric | 1 |
| 3 | Study site | 11=Bangladesh, 12=DRC, 13=Ghana, 15=India Shivgarh, 16=Kenya, 17=Pakistan Karachi, 18=Pakistan Matiari, 20=Tanzania Pemba, 21=Zambia | SITE | Numeric | 2 |
| 4 | Worker code | Each site to define valid range | FW | Text | 3 |
| 5 | Woman ID | Each site to define valid range | WHOWID | Text | 10 |
| 6 | Was the pregnant woman available for interview? | 11=present, 12=currently in hospital, 13=temporarily absent, 14=died, 15=permanently moved out, 16=refused temporarily, 17=withdrawn consent, 18=visit could not be made | AWSTATUS | Numeric | 2 |
|  | If visit could not be made, specify the reason | Text | NA_REASON | Text | 50 |
| 7 | If pregnant woman died, date of death | 01/06/2010 to 30/04/2013, 08/08/1908=don’t know, 09/09/1909=not applicable | AWDATEDIED | Date | 8 |
| 8 | Are you still pregnant? | 1=yes, 2=no, 8=don’t know, 9=not applicable | APREGNANT | Numeric | 1 |
| 9 | If no, what was the outcome of your recently reported pregnancy? | 11=miscarriage/abortion, 12= still birth, 13=live born baby alive, , 14=was not pregnant(false alarm/ previously falsely reported), 15=still pregnant, 88= don’t know, 99= not applicable (woman died) | AENDPREG | Numeric | 2 |
| 10 | If no longer pregnant, when did the recent pregnancy end? | 01/06/2010 to 30/04/2013 (sites to specify date range), 08/08/1908=don’t know, 09/09/1909=NA | ADATEDEL | Date | 8 |
| 11 | Which weeks of pregnancy is this? | Week 01 to 42, 88=don’t know, 99=NA | WKPRE | Numeric | 2 |
| 12 | Which months of pregnancy is this? | Month 01 to 10, 88=don’t know, 99=NA | MTHPREG | Numeric | 2 |
| **General Health** | | | | | |
| 13 | How would you rate your health today? | 11=excellent, 12=moderate, 13=poor | AGHEALTH | Numeric | 2 |
| **Hemorrhage** | | | | | |
| 14 | During this pregnancy / since the last visit, were you told that you had a hemorrhage? | 1=yes, 2=no, 8=don’t know | ATOLDHEMO | Numeric | 1 |
| 15 | During this pregnancy / since the last visit, have you had any bleeding from the vagina? | 1=yes, 2=no, 8=don’t know | ABLEED | Numeric | 1 |
| 16 | Did the bleeding last for longer than one day? | 1=yes, 2=no, 8=don’t know, 9=NA | ALBLEED | Numeric | 1 |
| 17 | Did the bleeding wet your clothes, the bed or the floor? | 1=yes, 2=no, 8=don’t know, 9=NA | ASEVBLEED | Numeric | 1 |
| 18 | Was the blood fresh (bright red) compared to dark red and viscous? | 1=fresh, 2=not fresh, 8=don’t know, 9=NA | AFRESHBLD | Numeric | 1 |
| 19 | Did you lose consciousness around the time of or because of the bleeding? | 1=yes, 2=no, 8=don’t know, 9=NA | ABLDCON | Numeric | 1 |
| **Hypertensive Disorders of Pregnancy** | | | | | |
| 20 | During this pregnancy / since the last visit, were you told that you have high blood pressure? | 1=yes, 2=no, 8=don’t know | ABPTOLD | Numeric | 1 |
| 21 | Measure systolic blood pressure and record results | xxx-yyy, 888=don’t know, 999=NA | ABPSYS | Numeric | 3 |
| 22 | Measure diastolic blood pressure and record results | xxx-yyy, 888=don’t know, 999=NA | ABPDIAS | Numeric | 3 |
| 23 | Measure proteinuria using dipstick and record result | 1=2+, 2=1+, 3=normal, 8=don’t know, 9=NA | ADIPSTICK | Numeric | 1 |
| 24 | During this pregnancy / since the last visit, did you have a headache? | 1=yes, 2=no, 8=don’t know | AHEAD | Numeric | 1 |
| 25 | For how long did you have a headache? | xx-yy days, 88=don’t know, 99=NA | ALHEAD | Numeric | 2 |
| 26 | Was the headache severe? | 1=yes, 2=no, 8=don’t know, 9=NA | ASEVHEAD | Numeric | 1 |
| 27 | During this pregnancy / since the last visit, did you have any swelling? | 1=yes, 2=no, 8=don’t know | ASWELL | Numeric | 1 |
| 28 | For how long did you have the swelling? | xx-yy days, 88=don’t know, 99=NA | ALSWELL | Numeric | 2 |
| 29 | Was the swelling on your whole body? | 1=yes, 2=no, 8=don’t know, 9=NA | ABDYSWELL | Numeric | 1 |
| 30 | Was the swelling on your joints? | 1=yes, 2=no, 8=don’t know, 9=NA | AJNTSWELL | Numeric | 1 |
| 31 | Was the swelling on your ankles? | 1=yes, 2=no, 8=don’t know, 9=NA | AANKSWELL | Numeric | 1 |
| 32 | Was the swelling on your face? (Did you have a puffy face?) | 1=yes, 2=no, 8=don’t know, 9=NA | AFACSWELL | Numeric | 1 |
| 33 | If the swelling was on any other part of the body, please specify where | Text | AOTHSWELL | Text | 15 |
| 34 | During this pregnancy / since the last visit, did you have blurred vision? | 1=yes, 2=no, 8=don’t know | AVISION | Numeric | 1 |
| 35 | For how long did you have blurred vision? | xx-yy days, 88=don’t know, 99=NA | ALVISION | Numeric | 2 |
| 36 | During this pregnancy / since the last visit have you had convulsions? | 1=yes, 2=no, 8=don’t know | ACONVULSE | Numeric | 1 |
| 37 | For how long did you have convulsions? | xx-yy days, 88=don’t know, 99=NA | ALCONVULS | Numeric | 1 |
| 38 | Did you lose consciousness because of the convulsions? | 1=yes, 2=no, 8=don’t know, 9=NA | ACONVCON | Numeric | 1 |
| **Fistula** | | | | | |
| 39 | During this pregnancy / since the last visit, did you experience continuously dripping urine? | 1=yes, 2=no, 8=don’t know | AURINEDRIP | Numeric | 1 |
| 40 | During this pregnancy / since the last visit, did you ever lose urine during sudden physical exertion, lifting, coughing or sneezing? | 1=yes, 2=no, 8=don’t know | AURINELOSE | Numeric | 1 |
| 41 | During this pregnancy / since the last visit, did you ever experience such a strong and sudden urge to urinate that you leak before reaching the toilet? | 1=yes, 2=no, 8=don’t know | AURINELEAK | Numeric | 1 |
| 42 | During this pregnancy / since the last visit, have you experienced feces passing through the birth canal? | 1=yes, 2=no, 8=don’t know | AFECESLEAK | Numeric | 1 |
| 43 | Has any physician or healthcare provider ever told you that you have a medical problem involving your vagina/pelvis or bladder while not pregnant? | 1=yes, 2=no, 8=don’t know | AVAGPROB | Numeric | 1 |
| 44 | Have you ever undergone surgery in the vagina/pelvis or bladder not including cesarean section, abortion, or surgery for contraception? | 1=yes, 2=no, 8=don’t know | AVAGSURG | Numeric | 1 |
| **Care Seeking** | | | | | |
| 45 | Were you in a facility when any of these symptoms occurred? | 1=yes, 2=no, 8=don’t know | AFACSYMP | Numeric | 1 |
| 46 | If you were at a facility when you experienced any of these symptoms, what type of facility was it? | 11=government hospital, 12=government clinic/health centre, 13=private hospital, 14=private clinic/health centre, 66=other, 88=don’t know, 99=NA | AFCTYPSYM | Numeric | 2 |
| 47 | If you were at a facility when you experienced any of these symptoms, were you referred to another facility? | 1=yes, 2=no, 8=don’t know, 9=NA | AFACREF | Numeric | 1 |
| 48 | If you were at a facility when you experienced any of these symptoms and were referred, what type of facility was it? | 11=government hospital, 12=government clinic/health centre, 13=private hospital, 14=private clinic/health centre, 66=other, 88=don’t know, 99=NA | AFCREFTYP | Numeric | 2 |
| 49 | If you were at a facility when you experienced any of these symptoms and were referred, did you go to the referral facility? | 1=yes, 2=no, 8=don’t know, 9=NA | AFACREFGO | Numeric | 1 |
| 50 | If you were not at a facility when you experience any of these symptoms, did you seek care? | 1=yes, 2=no, 9=NA | ASYMPSEEK | Numeric | 1 |
| 51 | If you sought care for any of the symptoms, for which symptoms did you seek care? | Bleeding: 1=yes, 2=no, 9=NA | ABLDSEEK | Numeric | 1 |
|  |  | High blood pressure: 1=yes, 2=no, 9=NA | AHBPSEEK | Numeric | 1 |
|  |  | Severe headache: 1=yes, 2=no, 9=NA | AHEADSEEK | Numeric | 1 |
|  |  | Swelling: 1=yes, 2=no, 9=NA | ASWELSEEK | Numeric | 1 |
|  |  | Blurred vision: 1=yes, 2=no, 9=NA | AVISSEEK | Numeric | 1 |
|  |  | Convulsions: 1=yes, 2=no, 9=NA | ACONVSEEK | Numeric | 1 |
|  |  | Loss of consciousness: 1=yes, 2=no, 9=NA | ALCSEEK | Numeric | 1 |
|  |  | Leakage of urine or feces: 1=yes, 2=no, 9=NA | ALEAKSEEK | Numeric | 1 |
| 52 | If you sought care, what was the first place where you sought care? | 11=government hospital, 12=government clinic/health centre, 13=private hospital, 14=private clinic/health centre, 15=community health worker, 16=traditional healer, 17=pharmacy/druggist,18=traditional birth attendant, 66=other, 99=NA | APLCCSEEK | Numeric | 2 |
| 53 | Where you admitted to sleep at a facility because of the symptoms you experienced? | 1=yes, 2=no, 9=NA | AADMIN | Numeric | 1 |
| 54 | For how many nights were you admitted? | 11=one day only, 12=1-3 days total, 13=4-6 days total, 14=more than 6 days total, 88=don’t know, 99=NA | ANUMADMIN | Numeric | 2 |
| 55 | If you sought care, did you receive any of the following treatments? | Hysterectomy (operation to remove your womb): 1=yes, 2=no, 9=NA | AHYSTEREC | Numeric | 1 |
|  |  | Laparotomy or other surgery (any operation where they put you to sleep and operated on your abdomen): 1=yes, 2=no, 9=NA | ASURGERY | Numeric | 1 |
|  |  | Blood transfusion: 1=yes, 2=no, 9=NA | ABLDTRANS | Numeric | 1 |
|  |  | Assisted breathing (by hand or by machine): 1=yes, 2=no, 9=NA | AVENTILATE | Numeric | 1 |
|  |  | IV fluid: 1=yes, 2=no, 9=NA | AIVFLUID | Numeric | 1 |
|  |  | Drugs/pills/medications: 1=yes, 2=no, 9=NA | ADRUGS | Numeric | 1 |
|  |  | Other treatment (specify) | AOTHTRTMT | Text | 30 |
| **MCH Document / ANC Card** | | | | | |
| 56 | Weight of woman (kg) | xx.x-yy.y, 99.9=NA | AFCWEIGHT | Numeric | 3 |
| 57 | Height of woman (cm) | xxx-yyy, 999=NA | AFCHEIGHT | Numeric | 3 |
| 58 | LMP | 01/08/2009 to 30/04/2013 (sites to specify date range), 08/08/1908=don’t know, 09/09/1909=NA | AFCLMP | Date | 8 |
| 59 | Number of antenatal visits | xx-yy, 99=NA | AFCNUMANC | Numeric | 2 |
| 60 | Estimated date of delivery | 01/08/2009 to 30/04/2013 (sites to specify date range), 08/08/1908=don’t know, 09/09/1909=NA | AFCDATDELIV | Date | 8 |
| 61 | Number of Admissions | xx-yy, 99=NA | FCNUMADM | Numeric | 2 |
| 62 | Treatments | Hysterectomy: 1=yes, 2=no, 9=NA | AFCHYSTER | Numeric | 1 |
|  |  | Laparotomy or other surgery: 1=yes, 2=no, 9=NA | AFCSRGY | Numeric | 1 |
|  |  | Blood transfusion: 1=yes, 2=no, 9=NA | AFCBLDTRN | Numeric | 1 |
|  |  | Assisted breathing: 1=yes, 2=no, 9=NA | AFCVENTIL | Numeric | 1 |
|  |  | IV fluid: 1=yes, 2=no, 9=NA | AFCIVFLUID | Numeric | 1 |
|  |  | Drugs/pills/medications: 1=yes, 2=no, 9=NA | AFCDRUGS | Numeric | 1 |
|  |  | Other treatment (specify) | AFCOTHER | Text | 30 |

| **POSTNATAL FORM (1-6 days after birth, 42-60 days after birth)** | | | | | |
| --- | --- | --- | --- | --- | --- |
| **No.** | **Question** | **Outcomes** | **Variable Name** | **Field Type** | **Size** |
| 1 | Date of filling the form  (all dates dd/mm/yyyy) | 01/06/2010 to 30/04/2013  (sites to specify date range) | P1DATEVISIT | Date | 8 |
| 2 | Study site | 11=Bangladesh, 12=DRC, 13=Ghana, 15=India Shivgarh, 16=Kenya, 17=Pakistan Karachi, 18=Pakistan Matiari, 20=Tanzania Pemba, 21=Zambia | SITE | Numeric | 2 |
| 3 | Worker code | Each site to define valid range | FW | Text | 3 |
| 4 | Woman ID | Each site to define valid range | WHOWID | Text | 10 |
| 5 | Did the mother deliver more than one child | 1=yes, 2=no | MULTI | Numeric | 1 |
| 6 | If yes, how many? | xx, 99=NA | NMULTI | Numeric | 1 |
| 7 | How many of these were live born? | Xx, 99=NA | LIVEMULTI | Numeric | 1 |
| 8 | Baby ID (only for live births) | Each site to define valid range, 99=NA | WHOBID | Text | 10 |
| 8.1 | Baby ID 2 (only for live births)  Continue with 8.2 and WHOBID3 etc. if needed | Each site to define valid range, 99=NA | WHOBID2 | Text | 10 |
| 9 | Live births/stillbirth:  Date of birth  Miscarriage/abortion:  Date of miscarriage/abortion | Each site to define valid range | P1DATEB | Date | 8 |
| 10 | Was the mother available for interview? | 11=present, 12=currently in hospital, 13=temporarily absent, 14=died, 15=permanently moved out, 16=refused temporarily, 17=withdrawn consent, 18=visit could not be made | P1WSTATUS | Numeric | 2 |
| 11 | If visit could not be made, specify the reason | Text | P1M_REASON | Text | 50 |
| 12 | If mother died, date of death | 01/06/2010 to 30/04/2013 (sites to specify date range), 08/08/1908=don’t know, 09/09/1909=NA | MDATEDIED | Date | 8 |
| 13 | Baby’s status at time of interview | Live births:  11=present, 12=currently in hospital, 13=temporarily absent, 14=died, 15=permanently moved out, 16=refused temporarily, 17=withdrawn consent, 18=visit could not be made  Other:  88=miscarriage/abortion, 99=baby stillborn | P1BSTATUS | Numeric | 2 |
| 13.1 | Baby 2’s status at time of interview  Continue with 13.2 and P1BSTATUS3 etc. if needed | Live births:  11=present, 12=currently in hospital, 13=temporarily absent, 14=died, 15=permanently moved out, 16=refused temporarily, 17=withdrawn consent, 18=visit could not be made  Other:  88=miscarriage/abortion, 99=baby stillborn | P1BSTATUS2 |  |  |
| 14 | If visit could not be made, specify the reason | Text | P1B_REASON | Text | 50 |
| 14.1 | If visit could not be made for baby 2, specify the reason  Continue with 14.2 and P1B_REASON3 etc. if needed | Text | P1B_REASON2 | Text | 50 |
| 15 | If baby died, date of death  (not applicable for miscarriage/abortion) | 01/06/2010 to 30/04/2013 (sites to specify date range), 08/08/1908=don’t know, 09/09/1909=NA | BDATEDIED | Date | 8 |
| 15.1 | If baby 2 died, date of death  Continue with 15.2 and BDATEDIED3 etc. if needed | 01/06/2010 to 30/04/2013 (sites to specify date range), 08/08/1908=don’t know, 09/09/1909=NA | BDATEDIED2 | Date | 8 |
| 16 | How many hours after birth did you first put the baby to the breast?  (not applicable for miscarriage/abortion) | 000 to 096, 888 = don’t know, 999= not applicable | P1BFSTART | Numeric | 3 |
| 16.1 | How many hours after birth did you first put baby 2 to the breast?  Continue with 16.2 and P1BFSTART3 etc. if needed | 000 to 096, 888 = don’t know, 999= not applicable | P1BFSTART2 | Numeric | 3 |
| **General Health** | | | | | |
| 17 | How would you rate your health today? | 11=excellent, 12=moderate, 13=poor | P1GHEALTH | Numeric | 2 |
| **Labor (only for live births or stillbirths)** | | | | | |
| 18 | When did your water break? | 1=before labor started, 2=after labor started or during delivery, 8=don’t know, 9=NA | P1WTRBRK | Numeric | 1 |
| 19 | How much time before delivery did the waters break? | 1=<24 hours, 2=24 hours or more, 8=don’t know, 9=NA (broke during delivery/did not break before C-section) | P1WTRBRKT | Numeric | 1 |
| 20 | Were the waters clear? | 1=clear, 2=not clear/dark, 8=don’t know, 9=NA (waters didn’t break) | P1WTRCLR | Numeric | 1 |
| 21 | How much time before delivery did your labor pains start? | 1=<24 hours, 2=24 hours or more, 8=don’t know, 9=NA | P1LBRSTART | Numeric | 1 |
| **Delivery (only for live births or stillbirths)** | | | | | |
| 22 | Where did the delivery take place? | 1=hospital, 2=other health facility, 3=home, 6=other (specify), 8=don’t know, 9=NA | P1PLCDELIV | Numeric | 1 |
| 23 | Who assisted the delivery | 1=doctor, 2=nurse/midwife, 3=TBA, 4=relative, 5=mother herself, 6=other (specify), 8=don’t know, 9=NA | P1ATNDNT | Numeric | 1 |
| 24 | What type of delivery was it? | 1=normal vaginal, 2=assisted vaginal (forceps/vacuum), 3=Cesarean section, 8=don’t know, 9=NA | P1TYPDELIV | Numeric | 1 |
| 25 | Which part of the baby came out first? | 1=head, 2=bottom, 3=feet, 4=arm/hand, 6=other (specify), 8=don’t know, 9=NA (C/S) | P1PRTDELIV | Numeric | 1 |
| 25.1 | For baby 2:  Which part of the baby came out first  Continue with 25.2 and P1PRTDELIV3 etc. if needed | 1=head, 2=bottom, 3=feet, 4=arm/hand, 6=other (specify), 8=don’t know, 9=NA (C/S) | P1PRTDELIV2 | Numeric | 1 |
| 26 | If vaginal delivery, did the umbilical cord come out before the baby? | 1=yes, 2=no, 8=don’t know, 9=NA | P1CORD | Numeric | 1 |
| 26.1 | For baby 2:  If vaginal delivery, did the umbilical cord come out before the baby?  Continue with 26.2 and P1CORD3 etc. if needed | 1=yes, 2=no, 8=don’t know, 9=NA | P1CORD2 | Numeric | 1 |
| 27 | Where was the cord when the baby came out? | 1=not around the baby, 2=around the neck, 3=around the body, 4=around other (specify), 8=don’t know, 9=NA | P1CORDPLC | Numeric | 1 |
| 27.1 | For baby 2:  Where was the cord when the baby came out?  Continue with 27.2 and P1CORDPLC3 etc. if needed | 1=not around the baby, 2=around the neck, 3=around the body, 4=around other (specify), 8=don’t know, 9=NA | P1CORDPLC2 | Numeric | 1 |
| 28 | Did the baby cry, move or breathe after birth? | 1=yes, 2=no, 8=don’t know, 9=NA | P1STILLB | Numeric | 1 |
| 28.1 | For baby 2:  Did the baby cry, move or breathe after birth?  Continue with 28.2 and P1STILLB3 etc. if needed | 1=yes, 2=no, 8=don’t know, 9=NA | P1STILLB2 | Numeric | 1 |
| 29 | If you had a cesarean section, did you know before you went into labor that you would have a cesarean section? | 1=yes, 2=no, 8=don’t know, 9=NA | P1KNOWCS | Numeric | 1 |
| 30 | If you had a cesarean section, is there pus coming from the scar? | 1=yes, 2=no, 8=don’t know, 9=NA | P1PUSCS | Numeric | 1 |
| 31 | If you had a cesarean section, was it because the baby was too big? | 1=yes, 2=no, 8=don’t know, 9=NA | P1CSBBIG | Numeric | 1 |
| 32 | If you had a cesarean section, was it because the baby was lying in a bad position? | 1=yes, 2=no, 8=don’t know, 9=NA | P1CSBPOS | Numeric | 1 |
| 33 | If you had a cesarean section, was it because you had a small pelvis? | 1=yes, 2=no, 8=don’t know, 9=NA | P1CSSMALL | Numeric | 1 |
| 34 | If you had a cesarean section, was it because you had a ruptured uterus or your uterus might rupture? | 1=yes, 2=no, 8=don’t know, 9=NA | P1CSURUPT | Numeric | 1 |
| 35 | If you had a cesarean section, was it because your uterus was too weak? | 1=yes, 2=no, 8=don’t know, 9=NA | P1CSUWEAK | Numeric | 1 |
| 36 | If you had a cesarean section, was it because of bleeding? | 1=yes, 2=no, 8=don’t know, 9=NA | P1CSBLEED | Numeric | 1 |
| 37 | If you had a cesarean section, was it because of convulsions? | 1=yes, 2=no, 8=don’t know, 9=NA | P1CSCONV | Numeric | 1 |
| 38 | Did the placenta come out within one hour of delivery? | 1=yes, 2=no, 8=don’t know, 9=NA | P1PLACENTA | Numeric | 1 |
| 39 | Did you have an episiotomy or did anyone cut your vagina during delivery? | 1=yes, 2=no, 8=don’t know, 9=NA | P1EPISIOT | Numeric | 1 |
| 40 | If yes, is there pus coming from the cut/tear? | 1=yes, 2=no, 8=don’t know, 9=NA | P1INFECEPIS | Numeric | 1 |
| **Hemorrhage (applicable for all)** | | | | | |
| 41 | Since the last visit, were you told that you had a hemorrhage? | 1=yes, 2=no, 8=don’t know | P1TLDHEMO | Numeric | 1 |
| 42 | Live birth/stillbirth:  If yes, were you told you had a hemorrhage before delivery?  Miscarriage/abortion:  If yes, were you told you had a hemorrhage before the miscarriage/abortion? | 1=yes, 2=no, 8=don’t know, 9=NA | P1TLDHEMBD | Numeric | 1 |
| 43 | Live birth/stillbirth:  If yes, were you told you had a hemorrhage during delivery?  Miscarriage/abortion:  If yes, were you told you had a hemorrhage during the miscarriage/ abortion? | 1=yes, 2=no, 8=don’t know, 9=NA | P1TLDHEMD | Numeric | 1 |
| 44 | Live birth/stillbirth:  If yes, were you told you had a hemorrhage after delivery?  Miscarriage/abortion:  If yes, were you told you had a hemorrhage after the miscarriage/ abortion? | 1=yes, 2=no, 8=don’t know, 9=NA | P1TLDHEMAD | Numeric | 1 |
| 45 | Since the last visit, have you had any bleeding from the vagina? | 1=yes, 2=no, 8=don’t know | P1BLEED | Numeric | 1 |
| 46 | Live birth/stillbirth:  If yes, did the bleeding from the vagina occur before delivery?  Miscarriage/abortion:  If yes, did the bleeding from the vagina occur before the miscarriage/ abortion? | 1=yes, 2=no, 8=don’t know, 9=NA | P1BLEEDBD | Numeric | 1 |
| 47 | Live birth/stillbirth:  If yes, did the bleeding from the vagina occur during delivery?  Miscarriage/abortion  If yes, did the bleeding from the vagina occur during the miscarriage/ abortion? | 1=yes, 2=no, 8=don’t know, 9=NA | P1BLEEDD | Numeric | 1 |
| 48 | Live birth/stillbirth:  If yes, did the bleeding from the vagina occur after delivery?  Miscarriage/abortion:  If yes, did the bleeding from the vagina occur after the miscarriage/ abortion? | 1=yes, 2=no, 8=don’t know, 9=NA | P1BLEEDAD | Numeric | 1 |
| 49 | Did the bleeding last for longer than one day? | 1=yes, 2=no, 8=don’t know, 9=NA | P1LBLEED | Numeric | 1 |
| 50 | Did the bleeding wet your clothes, the bed or the floor? | 1=yes, 2=no, 8=don’t know, 9=NA | P1SEVBLEED | Numeric | 1 |
| 51 | Was the blood fresh (bright red) compared to dark red and viscous? | 1=fresh, 2=not fresh, 8=don’t know, 9=NA | P1FRESHBLD | Numeric | 1 |
| 52 | Did you lose consciousness around the time of or because of the bleeding? | 1=yes, 2=no, 8=don’t know, 9=NA | P1BLDCON | Numeric | 1 |
| **Hypertensive Disorders of Pregnancy (applicable for all)** | | | | | |
| 53 | Since the last visit, were you told that you have high blood pressure? | 1=yes, 2=no, 8=don’t know | P1BPTOLD | Numeric | 1 |
| 54 | Measure systolic blood pressure and record results | xxx-yyy, 888=don’t know, 999=NA | P1BPSYS | Numeric | 3 |
| 55 | Measure diastolic blood pressure and record results | xxx-yyy, 888=don’t know, 999=NA | P1BPDIAS | Numeric | 3 |
| 56 | Measure proteinuria using dipstick and record result | 1=2+, 2=1+, 3=normal, 8=don’t know, 9=NA | P1DIPSTICK | Numeric | 1 |
| 57 | Since the last visit, did you have a headache? | 1=yes, 2=no, 8=don’t know | P1HEAD |  |  |
| 58 | For how long did you have a headache? | xx-yy days, 88=don’t know, 99=NA | P1LHEAD | Numeric | 2 |
| 59 | Was the headache severe? | 1=yes, 2=no, 8=don’t know, 9=NA | P1SEVHEAD | Numeric | 1 |
| 60 | Live birth/stillbirth:  If yes, did the severe headache occur before delivery?  Miscarriage/abortion:  If yes, did the severe headache occur before the miscarriage/ abortion? | 1=yes, 2=no, 8=don’t know, 9=NA | P1SHEADBD | Numeric | 1 |
| 61 | Live birth/stillbirth:  If yes, did the severe headache occur during delivery?  Miscarriage/abortion:  If yes, did the severe headache occur during the miscarriage/ abortion? | 1=yes, 2=no, 8=don’t know, 9=NA | P1SHEADD | Numeric | 1 |
| 62 | Live birth/stillbirth:  If yes, did the severe headache occur after delivery?  Miscarriage/abortion:  If yes, did the severe headache occur after the miscarriage/ abortion? | 1=yes, 2=no, 8=don’t know, 9=NA | P1SHEADAD | Numeric | 1 |
| 63 | Since the last visit, did you have any swelling? | 1=yes, 2=no, 8=don’t know | P1SWELL | Numeric | 1 |
| 64 | Live birth/stillbirth:  If yes, did you have swelling before delivery?  Miscarriage/abortion:  If yes, did you have swelling before the miscarriage/ abortion? | 1=yes, 2=no, 8=don’t know, 9=NA | P1SWELLBD | Numeric | 1 |
| 65 | Live birth/stillbirth:  If yes did you have swelling during delivery?  Miscarriage/abortion:  If yes did you have swelling during the miscarriage/ abortion? | 1=yes, 2=no, 8=don’t know, 9=NA | P1SWELLD | Numeric | 1 |
| 66 | Live birth/stillbirth:  If yes, did you have swelling after delivery?  Miscarriage/abortion:  If yes, did you have swelling after the miscarriage/ abortion? | 1=yes, 2=no, 8=don’t know, 9=NA | P1SWELLAD | Numeric | 1 |
| 67 | For how long did you have the swelling? | xx-yy days, 88=don’t know, 99=NA | P1LSWELL | Numeric | 2 |
| 68 | Was the swelling on your whole body? | 1=yes, 2=no, 8=don’t know, 9=NA | P1BDYSWELL | Numeric | 1 |
| 69 | Was the swelling on your joints? | 1=yes, 2=no, 8=don’t know, 9=NA | P1JNTSWELL | Numeric | 1 |
| 70 | Was the swelling on your ankles? | 1=yes, 2=no, 8=don’t know, 9=NA | P1ANKSWELL | Numeric | 1 |
| 71 | Was the swelling on your face? (Did you have a puffy face?) | 1=yes, 2=no, 8=don’t know, 9=NA | P1FACSWELL | Numeric | 1 |
| 72 | If the swelling was on any other part of the body, please specify where | Text | P1OTHSWELL | Text | 15 |
| 73 | Since the last visit, did you have blurred vision? | 1=yes, 2=no, 8=don’t know | P1VISION | Numeric | 1 |
| 74 | Live birth/stillbirth:  If yes, did you have blurred vision before delivery?  Miscarriage/abortion:  If yes, did you have blurred vision before the miscarriage/ abortion? | 1=yes, 2=no, 8=don’t know , 9=NA | P1VISIONBD | Numeric | 1 |
| 75 | Live birth/stillbirth:  If yes, did you have blurred vision during delivery?  Miscarriage/abortion:  If yes, did you have blurred vision during the miscarriage/ abortion? | 1=yes, 2=no, 8=don’t know , 9=NA | P1VISIOND | Numeric | 1 |
| 76 | Live birth/stillbirth:  If yes, did you have blurred vision after delivery?  Miscarriage/abortion:  If yes, did you have blurred vision after the miscarriage/ abortion? | 1=yes, 2=no, 8=don’t know , 9=NA | P1VISIONAD | Numeric | 1 |
| 77 | For how long did you have blurred vision? | xx-yy days, 88=don’t know, 99=NA | P1LVISION | Numeric | 2 |
| 78 | Since the last visit have you had convulsions? | 1=yes, 2=no, 8=don’t know | P1CONVULSE | Numeric | 1 |
| 79 | Live birth/stillbirth:  If yes, did you have convulsions before delivery?  Miscarriage/abortion:  If yes, did you have convulsions before the miscarriage/ abortion? | 1=yes, 2=no, 8=don’t know , 9=NA | P1CONVSBD | Numeric | 1 |
| 80 | Live birth/stillbirth:  If yes, did you have convulsions during delivery?  Miscarriage/abortion:  If yes, did you have convulsions during the miscarriage/ abortion? | 1=yes, 2=no, 8=don’t know , 9=NA | P1CONVD | Numeric | 1 |
| 81 | Live birth/stillbirth:  If yes, did you have convulsions after delivery?  Miscarriage/abortion:  If yes, did you have convulsions after the miscarriage/ abortion? | 1=yes, 2=no, 8=don’t know , 9=NA | P1CONVAD | Numeric | 1 |
| 82 | For how long did you have convulsions? | xx-yy days, 88=don’t know, 99=NA | P1LCONV | Numeric | 1 |
| 83 | Did you lose consciousness because of the convulsions? | 1=yes, 2=no, 8=don’t know, 9=NA | P1CONVCON | Numeric | 1 |
| **Sepsis (applicable for all)** | | | | | |
| 84 | Since my last visit, have you had a fever? | 1=yes, 2=no, 8=don’t know | P1FEVER | Numeric | 1 |
| 85 | Live birth/stillbirth:  If yes, did the fever occur before delivery?  Miscarriage/abortion:  If yes, did the fever occur before the miscarriage/ abortion? | 1=yes, 2=no, 8=don’t know, 9=NA | P1FEVERBD | Numeric | 1 |
| 86 | Live birth/stillbirth:  If yes, did the fever occur during delivery?  Miscarriage/abortion:  If yes, did the fever occur during the miscarriage/ abortion? | 1=yes, 2=no, 8=don’t know, 9=NA | P1FEVERD | Numeric | 1 |
| 87 | Live birth/stillbirth:  If yes, did the fever occur after delivery?  Miscarriage/abortion:  If yes, did the fever occur after the miscarriage/ abortion? | 1=yes, 2=no, 8=don’t know, 9=NA | P1FEVERAD | Numeric | 1 |
| 88 | If yes, did the fever last more than one day? | 1=yes, 2=no, 8=don’t know, 9=NA | P1LFEVER | Numeric | 1 |
| 89 | Since my last visit, did you have smelly discharge or pus pass from your vagina? | 1=yes, 2=no, 8=don’t know | P1VDISCHG | Numeric | 1 |
| 90 | If yes, when did the smelly discharge or pus pass from your vagina? | 1=before delivery, 2=after delivery, 8=don’t know, 9=NA | P1VDISCHGT | Numeric | 1 |
| 91 | Since the last visit, were you told that you have sepsis? | 1=yes, 2=no, 8=don’t know | P1SEPSIS | Numeric | 1 |
| 92 | If yes, when were you told that you have sepsis? | 1=before delivery, 2=after delivery, 8=don’t know, 9=NA | P1SEPSISTIM | Numeric | 1 |
| **Fistula (applicable for all)** | | | | | |
| 93 | Since the last visit, did you experience continuously dripping urine? | 1=yes, 2=no, 8=don’t know | P1URINDRIP | Numeric | 1 |
| 94 | Since the last visit, did you ever lose urine during sudden physical exertion, lifting, coughing or sneezing? | 1=yes, 2=no, 8=don’t know | P1URINLOSE | Numeric | 1 |
| 95 | Since the last visit, did you ever experience such a strong and sudden urge to urinate that you leak before reaching the toilet? | 1=yes, 2=no, 8=don’t know | P1URINLEAK | Numeric | 1 |
| 96 | Since the last visit, have you experienced feces passing through the birth canal? | 1=yes, 2=no, 8=don’t know | P1FECLEAK | Numeric | 1 |
| 97 | Since the last visit, has any physician or healthcare provider told you that you have a medical problem involving your vagina/pelvis or bladder while not pregnant? | 1=yes, 2=no, 8=don’t know | P1VAGPROB | Numeric | 1 |
| 98 | Since the last visit, have you undergone surgery in the vagina/pelvis or bladder not including cesarean section, abortion, or surgery for contraception? | 1=yes, 2=no, 8=don’t know | P1VAGSURG | Numeric | 1 |
| **Care Seeking (applicable for all)** | | | | | |
| 99 | Were you in a facility when any of these symptoms occurred? | 1=yes, 2=no, 8=don’t know, 9=NA | P1FSYMP | Numeric | 1 |
| 100 | If you were at a facility when you experienced any of these symptoms, what type of facility was it? | 11=government hospital, 12=government clinic/health centre, 13=private hospital, 14=private clinic/health centre, 66=other, 88=don’t know, 99=NA | P1FTYPSYM | Numeric | 2 |
| 101 | If you were at a facility when you experienced any of these symptoms, were you referred to another facility? | 1=yes, 2=no, 8=don’t know, 9=NA | P1FREF | Numeric | 1 |
| 102 | If you were at a facility when you experienced any of these symptoms and were referred, what type of facility was it? | 11=government hospital, 12=government clinic/health centre, 13=private hospital, 14=private clinic/health centre, 66=other, 88=don’t know, 99=NA | P1FREFTYP | Numeric | 2 |
| 103 | If you were at a facility when you experienced any of these symptoms and were referred, did you go to the referral facility? | 1=yes, 2=no, 8=don’t know, 9=NA | P1FREFGO | Numeric | 1 |
| 104 | If you were not at a facility when you experience any of these symptoms, did you seek care? | 1=yes, 2=no, 9=NA | P1SYMPSK | Numeric | 1 |
|  | If you sought care for any of the symptoms, for which symptoms did you seek care? | Bleeding: 1=yes, 2=no, 9=NA | P1BLDSK | Numeric | 1 |
|  |  | High blood pressure: 1=yes, 2=no, 9=NA | P1HBPSK | Numeric | 1 |
|  |  | Severe headache: 1=yes, 2=no, 9=NA | P1HEADSK | Numeric | 1 |
|  |  | Swelling: 1=yes, 2=no, 9=NA | P1SWELSK | Numeric | 1 |
|  |  | Blurred vision: 1=yes, 2=no, 9=NA | P1VSNSK | Numeric | 1 |
|  |  | Convulsions: 1=yes, 2=no, 9=NA | P1CONVSK | Numeric | 1 |
|  |  | Loss of consciousness: 1=yes, 2=no, 9=NA | P1LCSK | Numeric | 1 |
|  |  | Fever: 1=yes, 2=no, 9=NA | P1FVRSK | Numeric | 1 |
|  |  | Vaginal discharge/pus: 1=yes, 2=no, 9=NA | P1VAGDISK | Numeric | 1 |
|  |  | Leakage of urine or feces: 1=yes, 2=no, 9=NA | P1LEAKSK | Numeric | 1 |
| 105 | If you sought care, what was the first place where you sought care?? | 11=government hospital, 12=government clinic/health centre, 13=private hospital, 14=private clinic/health centre, 15=community health worker, 16=traditional healer, 17=pharmacy/druggist, 18=traditional birth attendant, 66=other, 99=NA | P1PLCCSK | Numeric | 2 |
| 106 | Where you admitted to sleep at a facility because of the symptoms you experienced? | 1=yes, 2=no, 9=NA | P1ADMIN | Numeric | 1 |
| 107 | For how many nights were you admitted? | 11=one day only, 12=1-3 days total, 13=4-6 days total, 14=more than 6 days total, 88=don’t know, 99=NA | P1NUMADM | Numeric | 2 |
| 108 | If you sought care, did you receive any of the following treatments? | Hysterectomy (operation to remove your womb): 1=yes, 2=no, 9=NA | P1HYSTER | Numeric | 1 |
|  |  | Laparotomy or other surgery (any operation where they put you to sleep and operated on your abdomen): 1=yes, 2=no, 9=NA | P1SURGERY | Numeric | 1 |
|  |  | Tear in vagina repaired: 1=yes, 2=no, 9=NA | P1REPTEAR | Numeric | 1 |
|  |  | Blood transfusion: 1=yes, 2=no, 9=NA | P1BLDTRANS | Numeric | 1 |
|  |  | Assisted breathing (by hand or by machine): 1=yes, 2=no, 9=NA | P1VENTIL | Numeric | 1 |
|  |  | IV fluid: 1=yes, 2=no, 9=NA | P1IVFLUID | Numeric | 1 |
|  |  | Drugs/pills/medications: 1=yes, 2=no, 9=NA | P1DRUGS | Numeric | 1 |
|  |  | Other treatment (specify) | P1OTHTRT | Text | 30 |
| **MCH Document / ANC Card** | | | | | |
| 109 | Weight of baby (gram)  (not applicable for miscarriage/abortion) | xxxx grams, 8888=DK, 9999=NA | P1BWEIGHT | Numeric | 4 |
| 109.1 | Weight of baby 2 (gram)  Continue with 109.2 and P1BWEIGHT3 etc. if needed | xxxx grams, 8888=DK, 9999=NA | P1BWEIGHT2 | Numeric | 4 |
| 110 | Weight of woman (kg) | xx.x-yy.y, 99.9=NA | P1FCWEIGHT | Numeric | 3 |
| 111 | Height of woman (cm) | xxx-yyy, 999=NA | P1FCHEIGHT | Numeric | 3 |
| 112 | LMP | 01/08/2009 to 30/04/2013 (sites to specify date range), 08/08/1908=don’t know, 09/09/1909=NA | P1FCLMP | Date | 8 |
| 113 | Number of antenatal visits | xx-yy, 99=NA | P1FCNUMANC | Numeric | 2 |
| 114 | Estimated date of delivery | 01/08/2009 to 30/04/2013 (sites to specify date range), 08/08/1908=don’t know, 09/09/1909=NA | P1FCDDELIV | Date | 8 |
| 115 | Number of Admissions | xx-yy, 99=NA | P1FCNUMADM | Numeric | 2 |
| 116 | Treatments | Hysterectomy: 1=yes, 2=no, 9=NA | P1FCHYSTER | Numeric | 1 |
|  |  | Laparotomy or other surgery: 1=yes, 2=no, 9=NA | P1FCSURG | Numeric | 1 |
|  |  | Blood transfusion: 1=yes, 2=no, 9=NA | P1FCBLDTRN | Numeric | 1 |
|  |  | Assisted breathing: 1=yes, 2=no, 9=NA | P1FCVENTIL | Numeric | 1 |
|  |  | IV fluid: 1=yes, 2=no, 9=NA | P1FCIVFLUID | Numeric | 1 |
|  |  | Drugs/pills/medications: 1=yes, 2=no, 9=NA | P1FCDRUGS | Numeric | 1 |
|  |  | Other treatment (specify) | P1FCOTHER | Text | 30 |
